# Development and validation of a novel risk prediction algorithm to estimate 10-year risk of oesophageal cancer in primary care: prospective cohort study and evaluation of performance against two other risk prediction models

**DOI:** 10.1016/j.lanepe.2023.100700

**Published:** 2023-08-14

**Authors:** Julia Hippisley-Cox, Winnie Mei, Rebecca Fitzgerald, Carol Coupland

**Affiliations:** a Nuffield Department of Primary Health Care Sciences, University of Oxford, England; b Early Cancer Institute, University of Cambridge and Addenbrooke's Hospital, Cambridge, England; c Centre for Academic Primary Care, School of Medicine, University Park, Nottingham, NG2 7R, England

**Keywords:** Oesophageal cancer, Early cancer detection, Prediction model, Targeted screening, Primary care

## Abstract

**Background:**

Methods to identify patients at increased risk of oesophageal cancer are needed to better identify those for targeted screening. We aimed to derive and validate novel risk prediction algorithms (CanPredict) to estimate the 10-year risk of oesophageal cancer and evaluate performance against two other risk prediction models.

**Methods:**

Prospective open cohort study using routinely collected data from 1804 QResearch® general practices. We used 1354 practices (12.9 M patients) to develop the algorithm. We validated the algorithm in 450 separate practices from QResearch (4.12 M patients) and 355 Clinical Practice Research Datalink (CPRD) practices (2.53 M patients). The primary outcome was an incident diagnosis of oesophageal cancer found in GP, mortality, hospital, or cancer registry data. Patients were aged 25–84 years and free of oesophageal cancer at baseline. Cox proportional hazards models were used with prediction selection to derive risk equations. Risk factors included age, ethnicity, Townsend deprivation score, body mass index (BMI), smoking, alcohol, family history, relevant co-morbidities and medications. Measures of calibration, discrimination, sensitivity, and specificity were calculated in the validation cohorts.

**Finding:**

There were 16,384 incident cases of oesophageal cancer in the derivation cohort (0.13% of 12.9 M). The predictors in the final algorithms were: age, BMI, Townsend deprivation score, smoking, alcohol, ethnicity, Barrett's oesophagus, hiatus hernia, *H. pylori* infection, use of proton pump inhibitors, anaemia, lung and blood cancer (with breast cancer in women). In the QResearch validation cohort in women the explained variation (R^2^) was 57.1%; Royston’s D statistic 2.36 (95% CI 2.26–2.46); C statistic 0.859 (95% CI 0.849–0.868) and calibration was good. Results were similar in men. For the 20% at highest predicted risk, the sensitivity was 76%, specificity was 80.1% and the observed risk at 10 years was 0.76%. The results from the CPRD validation were similar.

**Interpretation:**

We have developed and validated a novel prediction algorithm to quantify the absolute risk of oesophageal cancer. The CanPredict algorithms could be used to identify high risk patients for targeted screening.

**Funding:**

10.13039/501100006041Innovate UK and 10.13039/501100000289CRUK (grant 105857).

**Research in context**

**Evidence before this study**

Methods to identify patients at increased risk of oesophageal cancer are needed to identify patients for targeted screening, for example using non-endoscopic tools such as the Cytosponge-TFF3 test. There is no currently available tool to estimate 10-year risk of oesophageal cancer taking account of demographic, clinical and medication factors using information from primary care electronic records.

In preparation for ethical approval, we searched PubMed using the terms “oesophgeal cancer” AND “prediction model” in free text and Medical Subject Headings (MeSH) in “Title/Abstract” between January 1, 2000 and December 31, 2020, with no language restrictions, to understand the contemporary research on prediction models for oesophageal cancer and prepare a list of potential predictors for data specification.

**Added value of this study**

We have developed and externally validated a novel algorithm to quantify absolute risk of oesophageal cancer in men and women which is designed to work in primary care.

The algorithm provides valid measures of absolute risk in the general population of patients as shown by the performance in two separate validation cohorts, and comparison against two alternative prediction models.

**Implications of all the available evidence**

Further research is needed to evaluate the cost-effectiveness of using these algorithms in primary care to identify patients for further investigations or interventions.

## Introduction

Oesophageal cancer is the 8th most common cause of cancer worldwide.^1^ It continues to have a poor prognosis even in high income countries because most patients present with advanced disease when intensive multi-modal treatment is required with substantial effects on quality of life and poor outcomes.^2^

There is no systematic screening program for oesophageal cancer as the overall incidence in the general population is low (age standardised UK rate is 15 per 100,000^3^), although there are established risk factors for oesophageal cancer which could be used to develop a targeted screening approach in which patients are proactively offered diagnostic interventions such as endoscopy, including trans-nasal approaches^4^ or non-endoscopic tests such as Cytosponge-TFF3.^5^ Although targeted approaches are recommended^6^ and being tested as part of the new £6.4 million BEST4 trial,^7^ there is currently no validated predictive model available to identify those at high risk of oesophageal cancer in a primary care setting.^8^ Previous predictive models^9^, ^10^, ^11^ have been limited by small sample sizes, recall bias, failure to adjust for major risk factors such as age and sex.^8^ They are also histology specific models which limits their applications in the general population where oesophageal adenocarcinomas and squamous cell carcinomas co-exist.^12^ Other limitations include lack of granular information on predictor variables such as diagnosis of Barrett's oesophagus and hiatus hernia and use of prescribed medication.^9^

While both oesophageal adenocarcinoma (OAC) and squamous cell carcinoma (OSCC) are cancers of the oesophagus, they have different aetiologies hence there are some differences in risk factors. For example, previous studies have shown that lifestyle factors such as smoking and alcohol drinking are associated with an increased risk of both adenocarcinoma and squamous cell carcinoma. However, low socio-economic status was found to be associated with increased risk only for squamous cell carcinoma while obesity was associated with only adenocarcinoma. The incidence of oesophageal cancers in England also varies considerably by ethnic groups and by histology.^13^ Furthermore, there is emerging evidence that African Americans have a lower risk of adenocarcinoma compared to white Americans, despite having similar prevalence of gastric reflux disease, which is a major risk factor for adenocarcinoma.^14^

Electronic health records research databases from primary care linked to secondary care data contain routinely collected longitudinal population-based information as well as detailed information on predictor variables linked to cancer diagnosis. Such databases have been used to develop novel prediction algorithms such as QRISK3 to identify people at increased risk of cardiovascular disease which have then been implemented into NHS computer systems to support targeted cardiovascular disease prevention programmes such as the NHS Health Check in the UK.^15^

In this study, we used a large representative research database (QResearch) linked to cancer registrations, mortality and hospital episode statistics to develop and validate a novel predictive algorithm to better identify patients at high risk of oesophageal cancer over a ten-year period. We also externally validate the algorithm in a second external database, the Clinical Practice Research Datalink (CPRD) and compare with two alternative prediction algorithms.

## Methods

### Study design and data source

We undertook an open cohort study in a large population of primary care patients using the QResearch® database (version 45) and CPRD. QResearch is a large, representative, validated General Practice (GP) research database nationally. The database is linked at an individual patient level to hospital admissions, cancer registrations and mortality data and has been used extensively for the development of widely used risk prediction algorithms such as QRISK.^15^ The CPRD (Gold) database is similar but derived from GP practices using a different GP computer system (Vision) so represents a fully external cohort.

### Inclusion and exclusion criteria

We included GP practices contributing to the QResearch database using their Egton Medical Information System (EMIS) computer system for at least a year. We randomly allocated three quarters of practices to the derivation dataset and the remaining quarter to a validation dataset. We identified an open cohort of patients aged 25–84 years drawn from patients registered between 01 January 2005 and 31 March 2020. We excluded patients unsuitable for oesophageal cancer screening in primary care using non-endoscopic cell collection devices^5^ if they had any of the following at study entry: previous diagnosis of an oesophageal cancer or associated oesophageal surgical procedures; gastric or oral cancer; oesophageal varices and liver cirrhosis. We also excluded patients with a new onset of alarm symptoms (haematemesis, weight-loss or dysphagia) in the 90 days prior to cohort entry who are likely to require urgent referral. We also excluded patients without Townsend deprivation scores since these represent a very small proportion of the total population (usually <0.5%) where other important data such as sex and year of birth is frequently also missing so likely to represent incompletely registered patients.

Patients entered the cohort on the latest date of: 25th birthday, date of registration with the practice plus one year, date on which the practice computer system was installed plus one year, and the beginning of the study period (01 January 2005). Patients were categorised as having the outcome or censored at the earliest date of: recorded diagnosis of oesophageal cancer; death; deregistration with the practice; last upload of computerised data; or the study end date (31 March 2020). For the OAC model we censored people who had a diagnosis of OSCC at the date of diagnosis and vice versa.

We conducted a second validation using CPRD using the same criteria as above except the last date of follow up for which data were available was 1st January 2015.

### Primary outcome

Our primary outcome of interest was an incident diagnosis of primary oesophageal cancer during follow up in any of the four linked data sources (1) patient's GP record (2) linked mortality record (3) hospital record or (4) cancer registry record. Supplementary Table S1a lists the SNOMED-CT codes used to identify cases on the GP record and ICD-10 codes used to identify cases from the other data sources. We used the earliest recorded date of oesophageal cancer diagnosis on any of the four data sources as the index date. For patients where the only recorded diagnosis was on the death certificate, we used the date of death as a proxy for diagnosis.

The primary outcome for the CPRD database was the same except that only three data sources were used (GP, hospital and mortality) as linked cancer registry data were not available.

### Secondary outcome

Our secondary outcome was an incident diagnosis of adenocarcinoma and squamous cell carcinoma based on the histological type^2^ and ICD-10 location using information available on the linked cancer registry. See Supplementary Table S1b for further details. Data for the secondary outcome were only available for the QResearch database.

### Risk factors

We included established risk factors identified in the literature^8^^,^^16^ focusing on variables which are likely to be recorded in the patient's primary care electronic record. We did not include genetic markers since these are not yet routinely tested or recorded in primary care.

#### Demographic and lifestyle at or prior to cohort entry

• Age (in single years).
• Body mass index.
• Townsend deprivation score.
• Ethnicity (9 categories, white, Indian, Pakistani, Bangladeshi, Other Asian, Caribbean, Black African, Chinese, Other ethnic group).
• Smoking status - non-smoker; ex-smoker; light smoker (1–9/day); moderate smoker (10–19/day); heavy smoker (20+/day).
• Alcohol status—non-drinker; trivial (<1 unit/day); light (1–2 units/day); moderate (3–6 units/day); heavy (7–9 units/day); very heavy (>9 units/day).

#### Family history and medical conditions at or prior to cohort entry

• Barrett's oesophagus.
• Peptic ulcer disease.
•Type 1 and type 2 diabetes.
• Previous blood cancer.
• Previous lung cancer.
• Previous breast cancer (women).
• Previous pancreatic cancer.
• Previous Helicopter *pylori* (*H. pylori*) infection.
• Pernicious anaemia.
• Anaemia (most recent haemoglobin <110 g/L).
• Hiatus hernia.
• Gastro-oesophageal reflux.
• Family history of bowel cancer.

#### Medication use

We identified medications, based on the literature and expert opinion, which may be associated with oesophageal cancer either because they can increase the risk of it occurring or because they are used to treat associated symptoms.^17^ We categorised medication use according to the number of prescriptions ever issued prior to cohort entry for each of the following medications (categorised as no scripts, 1–5 scripts, 6–11 scripts, 12–23 scripts, 24–47 scripts, 48+ scripts).

• Proton pump inhibitors (PPI).
• H2 blockers.
• Non-steroidal anti-inflamatory drugs (NSAIDS).
• Aspirin.
• Statins.

### Model development

We developed and validated six risk prediction algorithms (known as the ‘CanPredict-oesophageal cancer algorithm) according to our pre-specified protocol.^17^ We developed sex specific equations for three outcomes - oesophageal cancer, OAC and OSCC.

We used Cox's proportional hazards models to estimate the coefficients for each risk factor for men and women separately using time since cohort entry as the underlying time function with follow up for up to 10 years. We used fractional polynomials^18^ to model non-linear risk relationships with continuous variables. We used multiple imputation with chained equations to replace missing values for ethnicity, body mass index, alcohol and smoking status and used these values in our main analyses.^19^ We carried out 5 imputations. We included all predictor variables in the imputation model, along with the Nelson–Aalen estimator of the baseline cumulative hazard, and the outcome indicator. We used Rubin's rules to combine the results across the imputed datasets**.** Where there was no recorded diagnosis or prescription, we assumed the patient did not have the relevant diagnosis or prescription.

We fitted full models and inspected the results. We then retained variables (including medications) in the final models that were statistically significant at the 1% level and where adjusted hazard ratios for binary variables were >1.1 or < 0.9 in accordance with our protocol and previous studies.^15^ We combined clinically similar variables with very low numbers of events. We examined interactions between predictor variables (including medications) and age. We assessed model optimism by calculating Van Houwelingen's measure of heuristic shrinkage.^20^ We used post estimation methods to estimate the baseline survivor function at 10 years from the Cox regression model based on zero values of centred continuous variables, with all binary predictor values set to zero. We used the mean of these values across all imputations in the calculation of predicted risks. Lastly, we combined regression coefficients from the final models with these estimates of the baseline survivor function evaluated at 10 years to derive separate risk equations for men and women for each outcome (six risk equations in total).

### Evaluation of model performance

We evaluated model performance in the QResearch and CPRD validation cohorts for overall oesophageal cancers. We used multiple imputation to replace missing values for ethnicity, BMI alcohol and smoking using the same imputation model as in the derivation cohort. We calculated Harrell's C statistic^21^ (measures of goodness-of-fit), Royston's R^2^ values^22^ (explained variation in time to diagnosis of oesophageal cancer), and the associated Royston's D statistics^23^ (measures of discrimination) at 10 years and combined these across imputed datasets using Rubin's rules. We conducted these analyses on each validation cohort.

Using both the QResearch and CPRD validation cohorts, we calculated the performance statistics across different subgroups for men and women (by ethnicity, age, region, quintile of Townsend deprivation score). We also evaluated performance by calculating Harrell's C statistics in individual general practices and combined the results using a random effects meta-analysis.^24^

We calculated Harrell's C statistics,^21^ Royston's R^2^ values and associated Royston's D statistics^22^ and combined them across imputed datasets using Rubin's rules.^25^ We ran a Cox proportional hazards model to calculate the calibration slope using the prognostic index over the study period. We assessed model calibration in both validation cohorts by calculating calibration slopes and calibration intercepts and compared mean predicted risks at 10 years with the observed risks by twentieths of predicted risk. We undertook a sensitivity analysis to evaluate calibration accounting for competing risk of non-oesophageal cancer death.^26^

We also compared model performance for two alternative score-based models including Kunzmann et al.^9^ and Wang et al.^27^ Details of these models can be found in Supplementary Table S2. In brief, the Kunzmann model was developed in a prospective cohort in the UK and predicted 5-year risk of OAC. The Wang model was developed to predict 5-year risk of OSCC using a Norwegian cohort and was validated in an UK cohort. For the comparisons, with the Kunzman score we used our OAC model and the OAC outcome and for the Wang score we used our OSCC model and the OSCC outcome.

### Decision curve analysis

We used decision curve analysis accounting for censoring in the validation cohort to evaluate the net benefits of the new risk equations.^28^ This approach assesses the benefits of correctly detecting people who will develop oesophageal cancer compared with the harms from a false positive classification (which could lead to unnecessary intervention). The net benefit of a risk equation at a given risk threshold is given by calculating the difference between the proportion of true positives and the proportion of false positives multiplied by the odds defined by the risk threshold value. We calculated the net benefits of using the algorithm across a range of threshold probabilities and compared these with alternative strategies such as assuming all patients are treated or no patients are treated. In general, the strategy with the highest net benefit at any given risk threshold is considered to have the most clinical value.

### Reporting

We adhered to the TRIPOD statements for reporting. We used all the available data on the database within the 75% sample to develop the models to maximise the power and generalisability of the results. We used Stata (version 17) for analyses.

### Sample size consideration

We used all the available data on the database to maximise the power and also generalisability of the results. We used STATA (version 17) for all analyses.

### Patient and public involvement and dissemination plans

Patients were involved in the grant application and steering groups. They have been invited to comment on the development of the model, its validation and potential implementation.

### Role of the funding source

The funder has no role in any role in study design, data collection, data analysis, interpretation, writing of the report.

## Results

### Study population

Overall, 1804 QResearch® practices in England met our inclusion criteria, of which 1354 were randomly assigned to the derivation cohort with the remaining 450 assigned to the validation cohort. Of the 13,053,500 patients aged 25–84 years in the derivation cohort, 125,348 were excluded leaving 12,928,152 (99%) for analysis. Of the 4,155,792 patients in the QResearch validation cohort, 38,265 were excluded leaving 4,117,527 (99.1%) in the analysis. Of the 3,294,988 patients in the CPRD validation cohort, 763,288 were excluded leaving 2,531,700 (76.8%) in the analysis. Supplementary Table S3a shows the numbers of patients with each exclusion criterion for both QResearch cohorts and the CPRD validation cohort. The larger number of exclusions in CPRD reflected the numbers of patients without a Townsend deprivation score.

Table 1 shows the baseline characteristics of men and women in the QResearch derivation, QResearch validation, and CPRD validation cohorts. It also shows the candidate variables assessed for model inclusion at baseline. In the derivation cohort, smoking status was recorded in 93.3%, alcohol intake in 80.9%, ethnicity in 72.2% and body mass index in 83.1%. These values were similar in the QResearch validation cohort. As in previous studies,^11^^,^^13^ the patterns of missing data supported the use of multiple imputation to replace missing values for these variables. Compared with QResearch cohort, the CPRD cohort was marginally older and had higher levels of missing data for ethnicity and tended to be more affluent.

**Table 1:** Baseline characteristics of patients in the QResearch derivation cohort, separate QResearch validation cohort, and CPRD validation cohort.

	QResearch derivation cohort	QResearch validation cohort	CPRD validation cohort
**Demographics**			
Total	12,928,152	4,117,527	2,531,700
Females	6,490,510 (50.2)	2,070,353 (50.1)	1,282,413 (50.7)
Males	6,437,642 (49.8)	2,047,174 (49.7)	1,249,287 (49.3)
Mean age (SD)	45.0 (15.6)	45.2 (15.6)	48.5 (15.3)
BMI recorded	10,739,993 (83.1)	3,442,087 (83.6)	1,863,301 (73.6)
Mean BMI (SD)	26.5 (5.3)	26.4 (5.2)	26.0 (4.7)
**Townsend quintile**^a^			
Quintile 1 (most affluent)	2,818,598 (21.8)	925,219 (22.5)	574,738 (22.7)
Quintile 2	2,639,993 (20.4)	866,434 (21.0)	567,695 (22.4)
Quintile 3	2,501,207 (19.3)	819,912 (19.9)	530,312 (20.9)
Quintile 4	2,434,628 (18.8)	773,474 (18.8)	502,354 (19.8)
Quintile 5 (most deprived)	2,533,726 (19.6)	732,488 (17.8)	356,601 (14.1)
**Ethnicity**			
White	7,440,292 (57.6)	2,352,271 (57.1)	1,047,638 (41.4)
Indian	336,729 (2.6)	121,469 (3.0)	30,928 (1.2)
Pakistani	197,842 (1.5)	60,925 (1.5)	12,308 (0.5)
Bangladeshi	138,408 (1.1)	35,150 (0.9)	3918 (0.2)
Other Asian	219,615 (1.7)	74,540 (1.8)	21,315 (0.8)
Caribbean	137,564 (1.1)	42,637 (1.0)	11,029 (0.4)
Black African	322,514 (2.5)	99,196 (2.4)	26,011 (1.0)
Chinese	103,559 (0.8)	27,559 (0.7)	6642 (0.3)
Other ethnic group	436,211 (3.4)	134,949 (3.3)	33,594 (1.3)
Ethnicity not recorded	3,595,418 (27.8)	1,168,831 (28.4)	1,338,317 (52.9)
**Region**			
East Midlands	459,366 (3.6)	198,196 (4.8)	87,161 (3.4)
East of England	681,896 (5.3)	223,256 (5.4)	306,841 (12.1)
London	3,645,420 (28.2)	1,092,930 (26.5)	411,037 (16.2)
North East	363,130 (2.8)	110,542 (2.7)	46,986 (1.9)
North West	2,054,377 (15.9)	567,813 (13.8)	360,448 (14.2)
South Central	1,621,502 (12.5)	372,490 (9.0)	326,058 (12.9)
South East	1,083,129 (8.4)	454,466 (11.0)	289,390 (11.4)
South West	1,221,189 (9.4)	510,163 (12.4)	314,132 (12.4)
West Midlands	1,258,991 (9.7)	414,422 (10.1)	273,382 (10.8)
Yorkshire & Humber	539,152 (4.2)	173,249 (4.2)	116,265 (4.6)
**Smoking status**			
Non-smoker	6,825,209 (52.8)	2,199,878 (53.4)	1,158,330 (45.8)
Ex-smoker	2,376,449 (18.4)	752,718 (18.3)	410,475 (16.2)
Light smoker (1–9/day)	2,134,924 (16.5)	668,557 (16.2)	387,933 (15.3)
Moderate smoker (10–19/day)	461,836 (3.6)	145,054 (3.5)	291,331 (11.5)
Heavy smoker (20+/day)	258,635 (2.0)	82,529 (2.0)	186,378 (7.4)
Smoking not recorded	871,099 (6.7)	268,791 (6.5)	97,253 (3.8)
**Alcohol status**			
Non-drinker	6,552,302 (50.7)	2,151,226 (52.2)	379,612 (15.0)
Trivial <1 units/day	2,006,819 (15.5)	656,845 (16.0)	963,440 (38.1)
Light 1–2 units/day	992,591 (7.7)	311,035 (7.6)	576,360 (22.8)
Moderate 3–6 units/day	796,232 (6.2)	262,295 (6.4)	168,747 (6.7)
Heavy 7–9u/day	61,847 (0.5)	19,992 (0.5)	18,980 (0.7)
Very Heavy >9 units/day	53,699 (0.4)	15,616 (0.4)	17,580 (0.7)
Alcohol status not recorded	2,464,662 (19.1)	700,518 (17.0)	406,981 (16.1)
**Current medication**			
H2 blockers	44,266 (0.3)	13,513 (0.3)	n/a
NSAID	188,422 (1.5)	58,463 (1.4)	n/a
Statins	487,226 (3.8)	155,377 (3.8)	n/a
Aspirin	262,060 (2.0)	81,039 (2.0)	n/a
**Proton pump inhibitors**			
No scripts	10,996,334 (85.1)	3,503,931 (85.1)	2,199,985 (86.9)
1–5 scripts	1,213,199 (9.4)	385,423 (9.4)	203,679 (8.0)
6–11 scripts	199,910 (1.5)	63,242 (1.5)	44,807 (1.8)
12–23 scripts	171,905 (1.3)	54,146 (1.3)	37,508 (1.5)
24–47 scripts	163,759 (1.3)	51,839 (1.3)	25,947 (1.0)
48+ scripts	183,045 (1.4)	58,946 (1.4)	19,774 (0.8)
**Medical conditions**			
Type 2 diabetes	509,547 (3.9)	163,115 (4.0)	n/a
Barrett's oesophagus	30,563 (0.2)	9580 (0.2)	5109 (0.2)
Blood cancer	36,772 (0.3)	11,767 (0.3)	6904 (0.3)
Breast cancer	86,635 (0.7)	28,563 (0.7)	20,857 (0.8)
Lung cancer	10,140 (0.1)	3334 (0.1)	1722 (0.1)
Peptic ulcer disease	244,881 (1.9)	77,580 (1.9)	65,650 (2.6)
Pernicious anaemia	23,309 (0.2)	7687 (0.2)	n/a
Hiatus hernia	246,741 (1.9)	78,847 (1.9)	55,050 (2.2)
Gastro-oesophageal reflux	610,338 (4.7)	191,333 (4.6)	n/a
H *pylori* infection	158,492 (1.2)	49,797 (1.2)	25,407 (1.0)

Values are numbers (%) unless stated otherwise).

a The Townsend score is an area-level score based on the patients' postcode which includes unemployment; non-car ownership and household overcrowding evaluated for a given area of approximately 120 households. A greater Townsend score implies a greater level of deprivation.

Table 2 shows the numbers of incident cases of oesophageal cancer during follow-up and incidence rates per 10,000 person-years in the QResearch derivation cohort. The same for the CPRD dataset can be found in Supplementary Table S3c. Supplementary Table S3d shows incidence rates by calendar year and Supplementary Table S3e shows rates by geographical region. Overall, the incidence rate was higher in CPRD compared with QResearch. Supplementary Table S4a shows the characteristics of incident oesophageal cancer cases. During follow up there were 16,384 cases of oesophageal cancer in the QResearch derivation cohort of which 11,535 cases (70.4%) occurred in men. The mean age at diagnosis was 69.6 (SD 10.7) years in men and 72.7 (SD 11.0) years in women in the derivation cohort. Age at diagnosis was similar in the QResearch validation cohort. Of the 16,384 cases in the derivation cohort, 6711 (41.0%) were adenocarcinoma, 3395 (20.7%) were squamous cell carcinoma; 640 (3.9%) were rare types and 5638 (34.4%) were unspecified. The results were similar for both the QResearch validation and CPRD validation cohorts. Supplementary Table S4b shows the number of cases diagnosed in each cohort by year since cohort entry.

**Table 2:** Number of cases of oesophageal cancer identified in the four linked data sources in QResearch derivation cohort and crude incidence rate per 10,000 person years.

Ageband	Identified on GP record		Identified on ONS mortality record		Identified on either GP or ONS or hospital record		Identified on either GP or ONS or hospital or cancer registry record	
	Cases	Rate per 10,000 person years	Cases	Rate per 10,000 person years	Cases	Rate per 10,000 person years	Cases	Rate per 10,000 person years
25–29	34	0.04 (0.03–0.05)	22	0.02 (0.02–0.03)	43	0.04 (0.03–0.06)	44	0.05 (0.03–0.06)
30–34	56	0.06 (0.04–0.08)	28	0.03 (0.02–0.04)	71	0.07 (0.06–0.09)	75	0.08 (0.06–0.10)
35–39	162	0.17 (0.15–0.20)	101	0.11 (0.09–0.13)	206	0.22 (0.19–0.25)	210	0.22 (0.20–0.26)
40–44	333	0.38 (0.34–0.42)	217	0.25 (0.21–0.28)	453	0.51 (0.47–0.56)	466	0.53 (0.48–0.58)
45–49	659	0.85 (0.79–0.92)	450	0.58 (0.53–0.64)	834	1.08 (1.01–1.16)	842	1.09 (1.02–1.17)
50–54	1116	1.68 (1.59–1.78)	796	1.20 (1.12–1.29)	1388	2.09 (1.98–2.20)	1425	2.15 (2.04–2.26)
55–59	1770	2.76 (2.63–2.89)	1291	2.01 (1.90–2.12)	2212	3.45 (3.31–3.60)	2248	3.51 (3.36–3.65)
60–64	1909	3.66 (3.50–3.83)	1503	2.88 (2.74–3.03)	2419	4.64 (4.45–4.82)	2463	4.72 (4.54–4.91)
65–69	1997	4.63 (4.43–4.83)	1622	3.76 (3.58–3.94)	2586	5.99 (5.77–6.23)	2620	6.07 (5.84–6.31)
70–74	1914	5.67 (5.43–5.93)	1650	4.89 (4.66–5.13)	2517	7.46 (7.18–7.76)	2570	7.62 (7.33–7.92)
75–79	1654	6.70 (6.39–7.04)	1533	6.21 (5.91–6.53)	2137	8.66 (8.30–9.04)	2181	8.84 (8.48–9.22)
80–84	896	6.76 (6.33–7.22)	911	6.87 (6.44–7.33)	1203	9.08 (8.58–9.60)	1240	9.36 (8.85–9.89)
Female	3603	0.96 (0.93–0.99)	3009	0.80 (0.77–0.83)	4736	1.26 (1.23–1.30)	4849	1.29 (1.26–1.33)
Male	8897	2.38 (2.33–2.43)	7115	1.90 (1.86–1.95)	11,333	3.03 (2.98–3.09)	11,535	3.08 (3.03–3.14)
**Total**	**12,500**	**1.67 (1.64**–**1.70)**	**10,124**	**1.35 (1.33**–**1.38)**	**16,069**	**2.15 (2.11**–**2.18)**	**16,384**	**2.19 (2.15**–**2.22)**

### Predictor variables

**Fig. 1:**
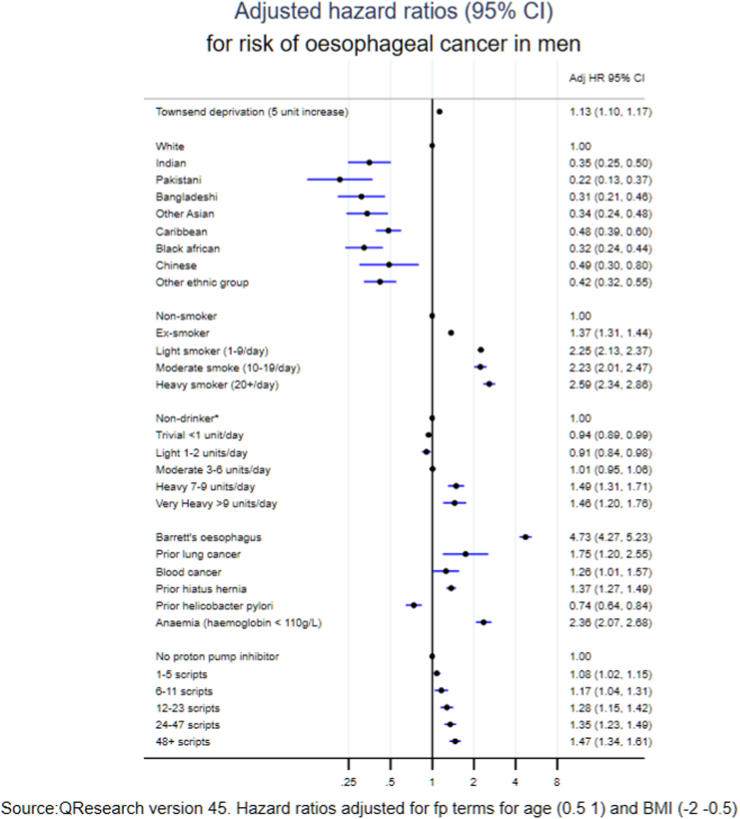
Adjusted hazard ratios (95% CI) for risk of oesophageal cancer in men.

**Fig. 2:**
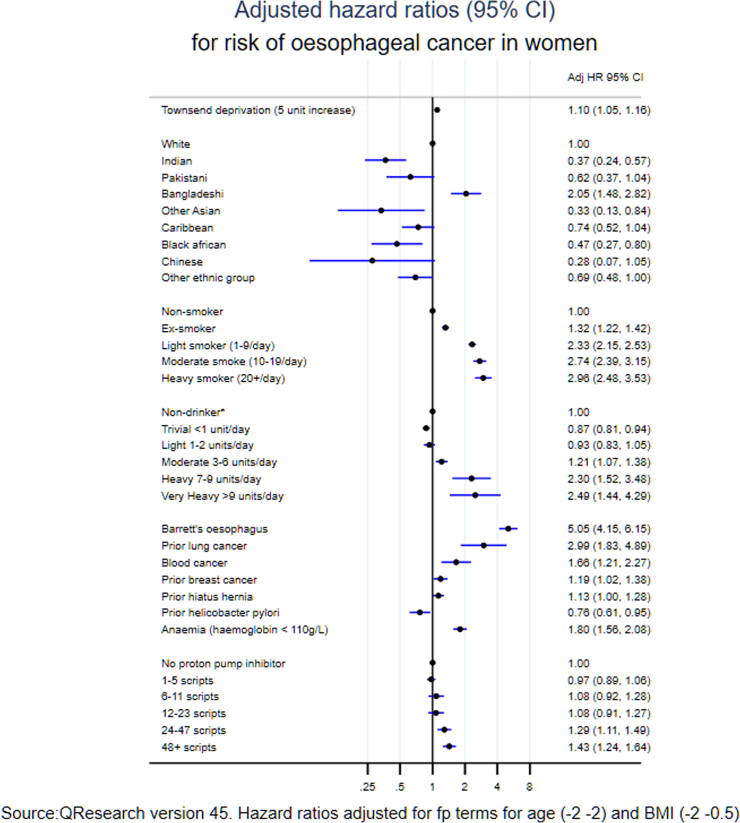
Adjusted hazard ratios (95% CI) for risk of oesophageal cancer in women.

**Supplementary Figure S1:**
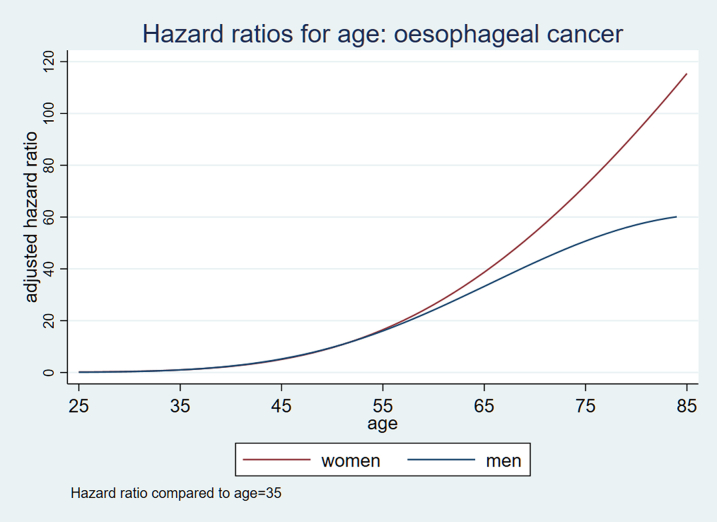


**Supplementary Figure S2:**
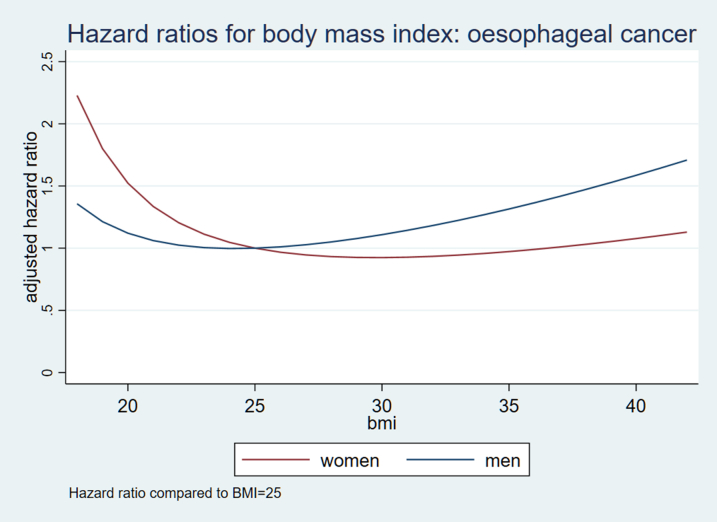


Fig. 1 shows the adjusted hazard ratios for variables in the final model for men (with adjusted hazard ratios for the fractional polynomial terms for age and body mass index in Supplementary Figures S1 and S2). The variables in the final model for oesophageal cancer in men were: age, body mass index, Townsend deprivation score, ethnic groups, smoking status (2.6-fold higher risk in heavy smokers compared with never smokers), alcohol (1.5-fold higher risk in heavy drinkers compared with non-drinkers), Barrett's oesophagus (4.7-fold higher risk), previous lung cancer (1.8-fold higher risk), previous blood cancer (1.3-fold higher risk), hiatus hernia (1.4-fold higher risk) and anaemia (2.4-fold higher risk). There was an increased risk associated with a higher number of PPI scripts (44% increased risk with 48 or more scripts of use compared with no scripts). There was a 26% lower risk of oesophageal cancer associated with prior *H. pylori* infection. The variables included in the final model for women (Fig. 2) were similar with an additional risk predictor of previous breast cancer (1.2-fold higher risk). The values for Van Houwelingen's heuristic shrinkage^20^ were very close to one (0.99 in women and men for all models). On the basis of our large sample size and these shrinkage values, we considered there was no need to modify the final model to take account of model optimism.

The risk of cancer varied by ethnic groups. For men, white ethnic group had higher risk than any other recorded ethnic groups. For women, Bangladeshi was the only ethnic group that was higher in risk (adjusted HR 2.05, 95% CI 1.48–2.82) than White; while all other ethnic groups were at lower risk than the White ethnic group. When considering oesophageal cancer overall we found no independent association for the following variables in either men or women: pernicious anaemia, indigestion, heartburn/reflux, oesophagitis, peptic ulcer disease, use of aspirin, NSAIDS, statins or H2 blockers.

**Supplementary Figure S3:**
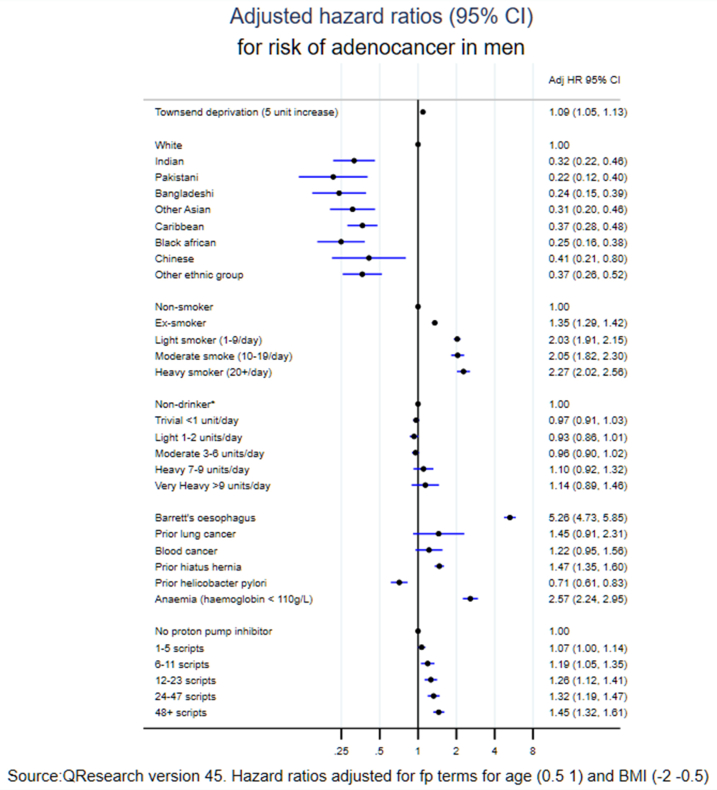


**Supplementary Figure S4:**
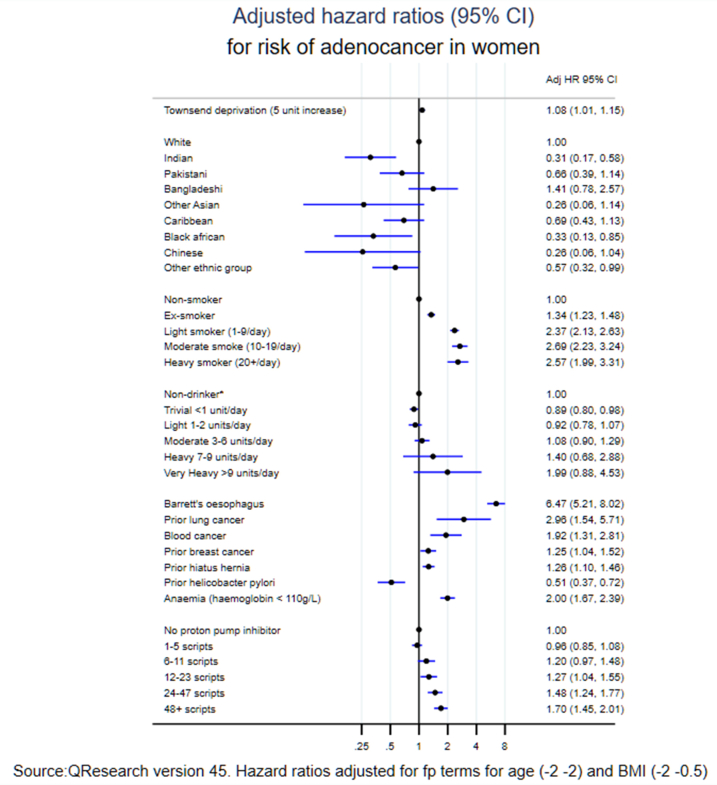


**Supplementary Figure S5:**
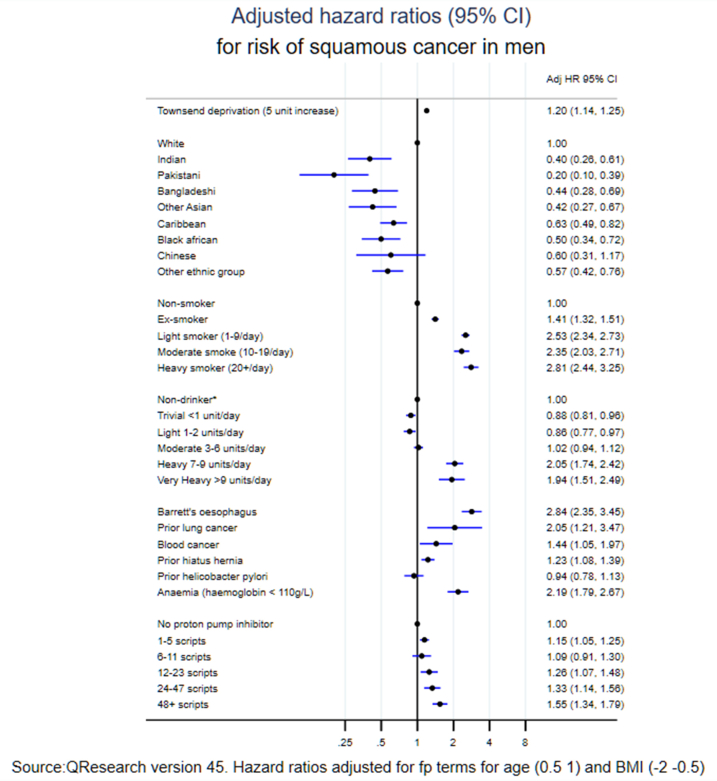


**Supplementary Figure S6:**
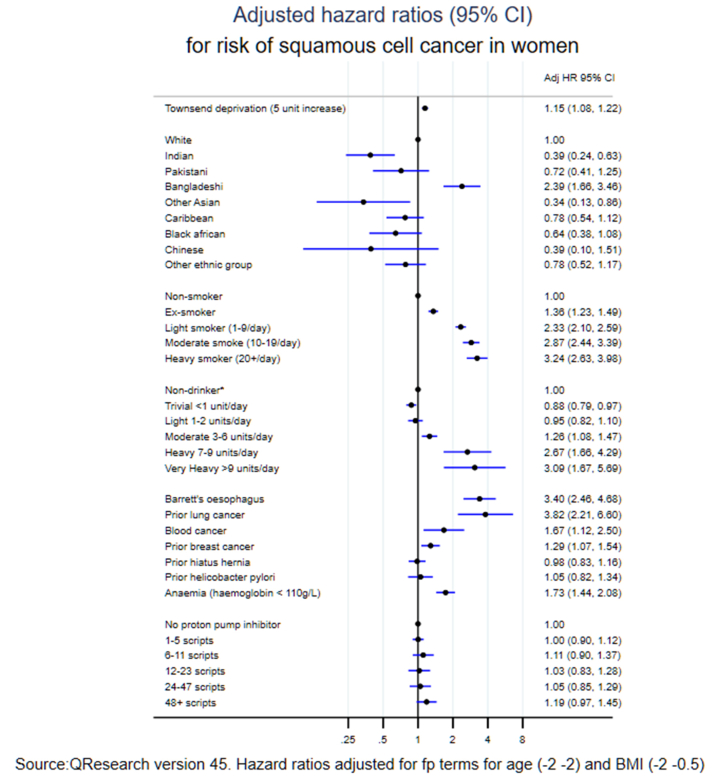


Supplementary Figures S3 and S4 show hazard ratios for men and women for oesophageal adenocarcinoma and Supplementary Figures S5 and S6 show the corresponding results for men and women for OSCC. Whilst the predictors in each model were the same, the weightings varied. As expected, the associations between Barrett's oesophagus and PPI prescriptions were more marked for oesophageal adenocarcinoma than squamous cell carcinoma. The associations with smoking and alcohol status were more marked for squamous cell carcinoma than adenocarcinoma. *H. pylori* infection was associated with a reduced risk of adenocarcinoma but no difference in risk of squamous cell carcinoma.

### Discrimination and calibration

Table 3 shows the overall performance of the oesophageal cancer risk prediction algorithm in the QResearch validation for women and men. The algorithm explained 57.1% (95% CI 55.0–59.1) of the variation in time to diagnosis in women (R^2^), Royston's D statistic was 2.36 (95% CI 2.26–2.46) and the Harrell's C statistic was 0.859 (95% CI 0.849–0.868). The corresponding results for men were marginally lower.

**Table 3:** Performance of the CanPredict risk algorithm in men and women aged 25–84 at baseline in the QResearch validation cohort and the CPRD validation cohort.

Statistics	QResearch validation cohort women	CPRD validation cohort women	QResearch validation cohort men	CPRD validation cohort men
	Mean (95% CI)	Mean (95% CI)	Mean (95% CI)	Mean (95% CI)
Harrell's C	0.859 (0.849–.868)	0.829 (0.818–0.839)	0.852 (0.846–0.858)	0.833 (0.827–0.84)
Royston's D statistic	2.36 (2.26–2.46)	2.11 (2.01–2.21)	2.23 (2.16–2.29)	2.08 (2.02–2.15)
R^2^ explained variation (%)	57.1 (55.0–59.1)	51.5 (49.2–53.8)	54.2 (52.8–55.7)	50.9 (49.4–52.4)

Performance measures for the model in the CPRD validation dataset were slightly lower but similar to the QResearch validation results for both men and women. In CPRD, the oesophageal cancer risk prediction algorithm explained 51.5% (95% CI 49.2–53.8) of the variation, with Royston's D statistic of 2.11 (95% CI 2.01–2.21) and Harrell's C of 0.829 (95% CI 0.818–0.839) in women.

The results for the performance of the other risk scores are shown in Table 4 (men) and 5 (women). Evaluation of the Kunzmann score was restricted to individuals aged 50–84 years and for Wang was restricted to those aged 40–84 years to match the cohort age range used in development of the respective scores. Overall the Wang score performed better in women and Kunzmann score fitted better in men. Wang score had higher discrimination (Royston's D statistics: 1.01 (95% CI 0.928–1.10) and explained more variation (R^2^: 19.7 (95% CI 17–22.3)) than the Kunzmann score (Royston's D statistics: 0.828 (95% CI 0.739–0.916), R^2^: in women. The overall variance explained by the Kunzmann model was 14.1% (95% CI 11.5–16.6)) in women and 16.4% (95% CI 14.6–18.2) in men; compared with 19.7% (95% CI 17–22.3) in women and 14.2% (95% CI 12.5–15.8) in men by Wang score. Tables 4 and 5 also show the comparison of the model performance between the other scores and the CanPredict algorithm, with the CanPredict algorithm fitted on the same subset of the individuals (I.e. in individuals aged 50–84 years for Kunzmann and 40–84 years for Wang) as the other two scores. To be consistent with the Kunzmann score, we generated a score which predicted risk of cancer at 5 years and evaluated its performance in QResearch validation cohort. Overall, the new oesophageal cancer risk prediction algorithm performed better than the external models irrespective of the age range or the predicted risk period.

**Table 4:** Performance of the Kunzmann, Wang and CanPredict risk algorithms in men in the QResearch validation cohort overall and by ethnic group.^9^^,^^10^

Statistic	Age 50–84 years		Age 40–84 years	
	5-year risk estimate Kunzmann (OAC)	5-year risk estimate CanPredict (OAC)	10-year risk estimate Wang (OSCC)	10-year risk estimate CanPredict (OSCC)
	Mean (95% CI)	Mean (95% CI)	Mean (95% CI)	Mean (95% CI)
**Harrell's C**				
**Overall**	0.665 (0.655–0.675)	0.699 (0.686–0.713)	0.649 (0.64–0.657)	0.771 (0.762–0.779)
White	0.663 (0.658–0.667)	0.702 (0.696–0.708)	0.642 (0.638–0.646)	0.769 (0.765–0.772)
Indian	0.711 (0.676–0.747)	0.693 (0.653–0.732)	0.688 (0.656–0.72)	0.781 (0.748–0.815)
Pakistani	0.689 (0.617–0.761)	0.684 (0.614–0.753)	0.6 (0.535–0.666)	0.713 (0.65–0.777)
Bangladeshi	0.465 (0.337–0.592)	0.595 (0.443–0.746)	0.596 (0.533–0.659)	0.546 (0.454–0.637)
Other Asian	0.572 (0.511–0.633)	0.57 (0.5120–0.629)	0.665 (0.623–0.708)	0.734 (0.693–0.776)
Caribbean	0.592 (0.548–0.635)	0.638 (0.59–0.687)	0.652 (0.61–0.694)	0.739 (0.703–0.776)
Black African	0.632 (0.574–0.689)	0.598 (0.526–0.67)	0.674 (0.627–0.721)	0.753 (0.711–0.796)
Chinese	0.709 (0.62–0.797)	0.707 (0.572–0.842)	0.687 (0.61–0.763)	0.849 (0.783–0.915)
Other	0.612 (0.569–0.655)	0.604 (0.554–0.654)	0.641 (0.595–0.686)	0.753 (0.71–0.797)
**Royston'sD statistic**				
**Overall**	0.905 (0.846–0.965)	1.19 (1.11–1.27)	0.832 (0.778–0.886)	1.63 (1.56–1.69)
White	0.89 (0.826–0.954)	1.2 (1.11–1.28)	0.795 (0.739–0.851)	1.58 (1.52–1.65)
Indian	1.22 (.718–1.72)	1.41 (.614–2.21)	1.06 (.453–1.67)	1.87 (1.21–2.52)
Pakistani	a	a	a	1.05 (.0451–2.05)
Bangladeshi	a	a	a	a
Other Asian	a	a	0.802 (0.052–1.55)	1.41 (.377–2.44)
Caribbean	0.766 (0.0452–1.49)	0.926 (0.025–1.83)	1.15 (.617–1.69)	1.39 (.744–2.04)
Black African	a	a	a	1.77 (.46–3.07)
Chinese	a	a	a	a
Other	a	0.897 (0.085–1.71)	0.846 (0.252–1.44)	1.63 (.918–2.34)
**R2 (%)**				
**Overall**	16.4 (14.6–18.2)	25.3 (22.6–27.9)	14.2 (12.6–15.8)	38.7 (36.9–40.6)
White	15.9 (14–17.8)	25.5 (22.8–28.2)	13.1 (11.5–14.7)	37.5 (35.5–39.5)
Indian	26.3 (10.3–42.2)	32.1 (7.78–56.5)	21.3 (2.26–40.4)	45.3 (28–62.6)
Pakistani	a	a	a	a
Bangladeshi	a	a	a	a
Other Asian	a	a	a	32 (.292–63.6)
Caribbean	a	a	24.1 (7.12–41.2)	31.7 (11.6–51.9)
Black African	a	a	a	42.1 (4.64–79.6)
Chinese	a	a	a	a
Other	a	a	a	38.6 (18.1–59.2)

a Cells with too few observationsto be estimated reliably.

**Table 5:** Performance of the Kunzmann, Wang and CanPredict risk algorithms in women in the QResearch validation cohort overall and by ethnic group.^9^^,^^10^

Statistic	5-year risk estimate Kunzmann (OAC) (age 50–84 years)	5-year risk estimate CanPredict (OAC) (age 50–84 years)	10-year risk estimate Wang (OSCC) (40–84 year)	10-year risk estimate CanPredict (OSCC) (40–84 year)
	Mean (95% CI)	Mean (95% CI)	Mean (95% CI)	Mean (95% CI)
**Harrell's C**				
**Overall**	0.649 (0.634–0.664)	0.725 (0.707–0.742)	0.679 (0.666–0.691)	0.79 (778–801)
White	0.643 (0.636–0.65)	0.722 (0.714–0.73)	0.663 (0.658–0.669)	0.785 (0.779–0.79)
Indian	0.746 (0.71–0.781)	0.769 (0.712–0.826)	0.793 (0.764–0.823)	0.873 (0.842–0.903)
Pakistani	0.603 (0.504–0.701)	0.684 (0.495–0.874)	0.711 (0.645–0.778)	0.748 (0.602–0.895)
Bangladeshi	0.627 (0.52–0.733)	0.816 (0.73–0.903)	0.773 (0.701–0.845)	0.799 (0.728–0.87)
Other Asian	0.607 (0.521–0.694)	0.522 (0.461–0.583)	0.753 (0.706–0.801)	0.75 (0.694–0.806)
Caribbean	0.626 (0.577–0.674)	0.82 (0.78–0.859)	0.78 (0.755–0.804)	0.817 (0.785–0.849)
Black African	0.779 (0.719–0.839)	0.81 (0.743–0.878)	0.731 (0.666–0.796)	0.815 (0.755–0.875)
Chinese	0.788 (0.714–0.862)	0.662 (0.527–0.797)	0.703 (0.607–0.799)	0.832 (0.74–0.924)
Other	0.786 (0.72700.845)	0.866 (0.815–0.917)	0.818 (0.778–0.858)	0.871 (0.823–0.92)
**Royston'sD statistic**				
**Overall**	0.828 (0.739–0.916)	1.45 (1.33–1.58)	1.01 (0.928–1.1)	1.76 (1.66–1.86)
White	0.798 (0.706–0.89)	1.45 (1.31–1.58)	0.942 (0.856–1.03)	1.71 (1.61–1.81)
Indian	1.35 (.604–2.09)	2.09 (1.06–3.11)	1.64 (0.899–2.38)	2.53 (1.65–3.41)
Pakistani	a	a	a	a
Bangladeshi	a	a	a	1.75 (0.00222–3.49)
Other Asian	a	a	1.27 (0.331–2.21)	1.7 (0.701–2.7)
Caribbean	a	1.79 (0.83–2.76)	1.41 (0.682–2.14)	1.8 (0.989–2.61)
Black African	a	2.04 (0.321–3.75)	a	a
Chinese	a	a	a	a
Other	a	2.89 (1.13–4.64)	2.05 (.227–3.87)	2.97 (1.22–4.71)
**R2 (%)**				
**Overall**	14.1 (11.5–16.6)	33.6 (29.7–37.4)	19.7 (17–22.3)	42.5 (39.8–45.2)
White	13.2 (10.6–15.8)	33.3 (29.3–37.4)	17.5 (14.8–20.1)	41.2 (38.3–44.1)
Indian	30.2 (6.98–53.5)	50.9 (26.2–75.5)	39 (17.6–60.5)	60.3 (43.6–77.1)
Pakistani	a	a	a	a
Bangladeshi	a	a	a	a
Other Asian	a	a	a	40.9 (12.4–69.4)
Caribbean	a	43.3 (16.9–69.8)	32.2 (9.79–54.6)	43.5 (21.4–65.7)
Black African	a	49.5 (5.76–93.2)	a	a
Chinese	a	a	a	a
Other	a	65.9 (39.4–92.4)	48.7 (6.96–90.4)	67 (43.3–90.7)

a Cells with too few observations to be estimated reliably.

Comparison of the performance measures between our model and external models by age band are shown in Supplementary Table S5. Performance statistics were higher for the new oesophageal cancer risk prediction algorithm compared with the other two scores in all age bands. All models performed consistently better in the younger age group (i.e. 25–49 for CanPredict or 40–49 in Wang) than the older groups.

The Harrell's C statistics were 0.739 (95% CI 0.721–0.757) for the CanPredict algorithm in people aged 50–59 years; compared with 0.723 (95% CI 0.708–0.739) for Kunzmann model; and 0.595 (95% CI 0.574–0.616) in Wang model.

Royston's D statistics in people aged 50–59 years were 1.49 (95% CI 1.38–1.61) in CanPredict algorithm; compared with 1.23 (95% CI 1.13–1.33) and 0.509 (95% CI 0.409–0.61) in Kunzmann and Wang models respectively.

The explained variation (R^2^) values in the age group of 50–59 years were 34.8% (95% CI 31.4–38.2), 26.6% (95% CI 23.5–29.6), 5.84% (95% CI 3.68–8.00) for CanPredict, Kunzmann, and Wang prediction algorithms respectively.

### Calibration

Table 6 shows values of the calibration slope are close to 1 and the calibration intercept values are close to zero, except in women in CPRD where the calibration intercept value of −0.42 indicates a degree of overestimation of risk.

**Table 6:** Calibration slope and intercept with 95% confidence intervals of the CanPredict algorithms in the QResearch and CPRD validation cohorts (ages 25–84 years).

	QResearch	CPRD
Men slope	0.967 (0.934–0.998)	1.045 (1.007–1.083
Men intercept	−0.034 (−0.066 to −0.002)	0.027 (−0.012 to 0.0699)
Women slope	0.992 (0.944–1.039)	0.965 (0.913–1.018)
Women intercept	−0.0082 (−0.056 to 0.039)	−0.421 (−1.04 to 0.194)

**Table 7:** Numbers of cases, sensitivity, specificity, and observed 10-year risk identified in each twentieth of predicted risk using the CanPredict algorithm in the QResearch validation cohort. Of 4,117,527 patients, 5014 developed oesophageal cancer during follow-up.

Top centile	Threshold of predicted 10-year risk (%)	Below threshold/no cancer^a^	Below threshold/cancer^b^	Above threshold/no cancer^c^	Above threshold/cancer^d^	Sensitivity%	Specificity%	Observed 10-year risk %
Top 5%	0.97	3,908,150	3501	204,363	1513	30.18	95.03	1.44 (1.35, 1.53)
Top 10%	0.63	3,703,315	2460	409,198	2554	50.94	90.05	1.09 (1.04, 1.14)
Top 15%	0.44	3,498,143	1755	614,370	3259	65.00	85.06	0.89 (0.85, 0.93)
Top 20%	0.31	3,292,817	1205	819,696	3809	75.97	80.07	0.76 (0.73, 0.78)
Top 25%	0.22	3,087,333	813	1,025,180	4201	83.79	75.07	0.65 (0.63, 0.67)
Top 30%	0.16	2,881,714	555	1,230,799	4459	88.93	70.07	0.57 (0.55, 0.59)
Top 35%	0.11	2,676,016	377	1,436,497	4637	92.48	65.07	0.50 (0.48, 0.52)
Top 40%	0.08	2,470,282	235	1,642,231	4779	95.31	60.07	0.45 (0.43, 0.46)
Top 45%	0.05	2,264,481	159	1,848,032	4855	96.83	55.06	0.40 (0.39, 0.42)
Top 50%	0.04	2,058,662	102	2,053,851	4912	97.97	50.06	0.37 (0.36, 0.38)
Top 55%	0.02	1,852,824	64	2,259,689	4950	98.72	45.05	0.34 (0.33, 0.35)
Top 60%	0.017	1,646,966	45	2,465,547	4969	99.10	40.05	0.31 (0.30, 0.32)
Top 65%	0.012	1,441,103	32	2,671,410	4982	99.36	35.04	0.29 (0.28, 0.30)
Top 70%	0.008	1,235,237	22	2,877,276	4992	99.56	30.04	0.28 (0.27, 0.29)
Top 75%	0.006	1,029,367	15	3,083,146	4999	99.70	25.03	0.26 (0.25, 0.27)
Top 80%	0.004	823,497	9	3,289,016	5005	99.82	20.02	0.25 (0.24, 0.26)
Top 85%	0.003	617,624	6	3,494,889	5008	99.88	15.02	0.24 (0.23, 0.25)
Top 90%	0.002	411,748	5	3,700,765	5009	99.90	10.01	0.23 (0.22, 0.24)
Top 100%	na	0	0	4,112,513	5014	100.0	0	0.22 (0.21, 0.23)

a Observed number of individuals below the risk threshold who did not develop oesophageal cancer.

b Observed number of individuals below the risk threshold who did develop oesophageal cancer.

c Observed number of individuals above the risk threshold who did not develop oesophageal cancer.

d Observed number of individuals above the risk threshold who did develop oesophageal cancer.

**Fig. 3:**
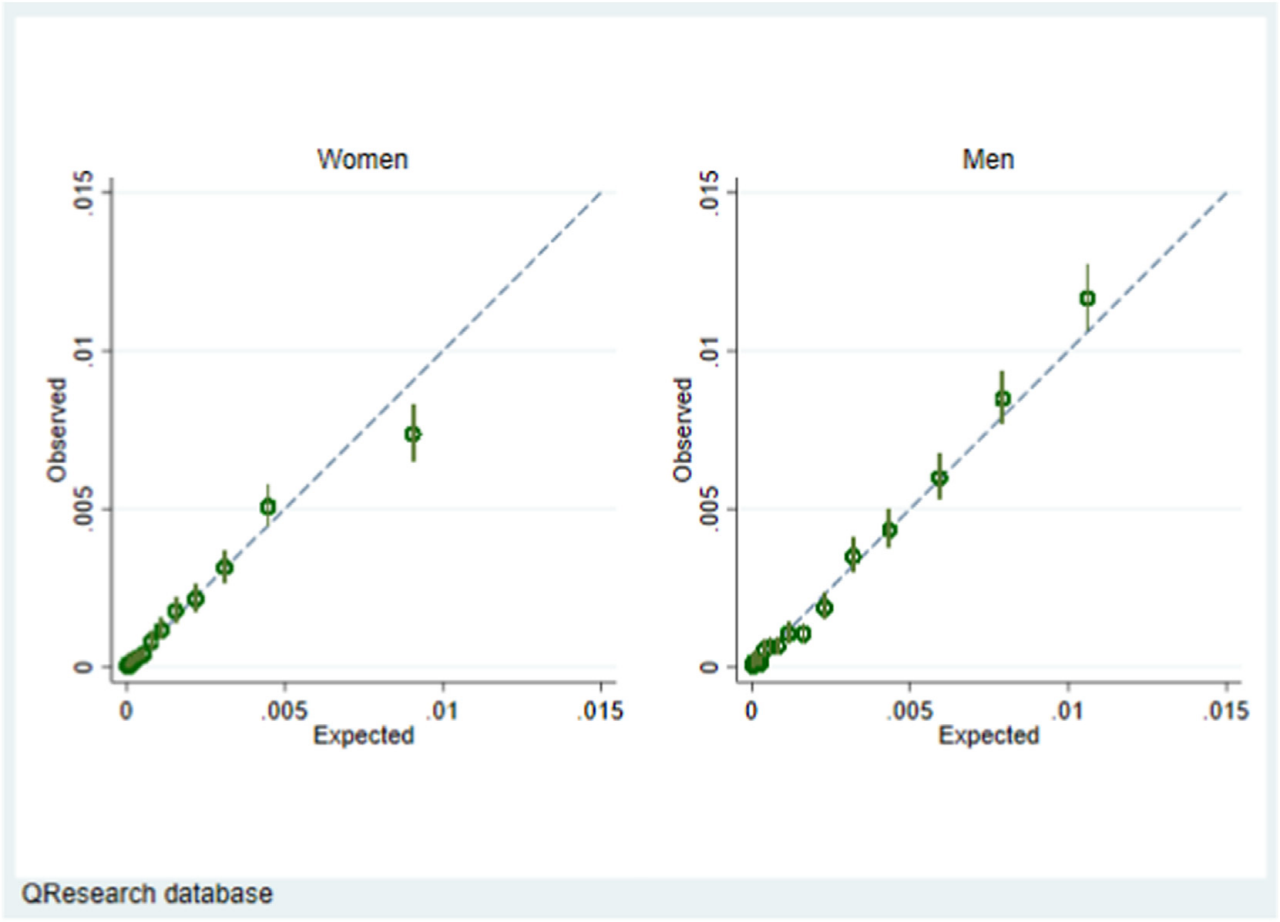
Calibration of the CanPredict algorithm model in men and women in the QResearch validation cohort.

Fig. 3 shows calibration of the CanPredict algorithm model in men and women in the QResearch validation cohort. There was close correspondence between the mean predicted risks and the observed risks within each model twentieth in women and men which indicates the algorithms are well calibrated except for a small degree of over prediction in the highest 5% for women. Supplementary Figure S19 displays calibration plots for our main model, evaluated (a) ignoring completing risks of non-oesophageal cancer death (Kaplan Meier estimates shown in red) and (b) using a competing risk approach (cumulative incidence function, shown in green). It shows some overprediction at highest levels of predicted risk in both men and women and this is more marked when competing risks are accounted for.

**Supplementary Figure S7:**
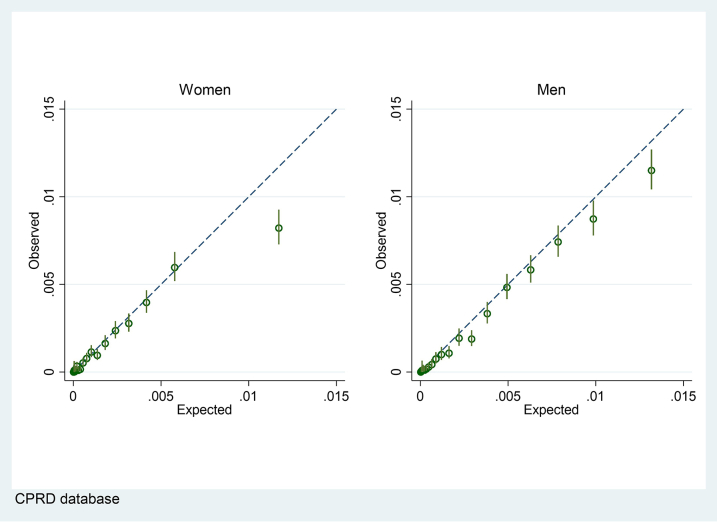


**Supplementary Figure S8:**
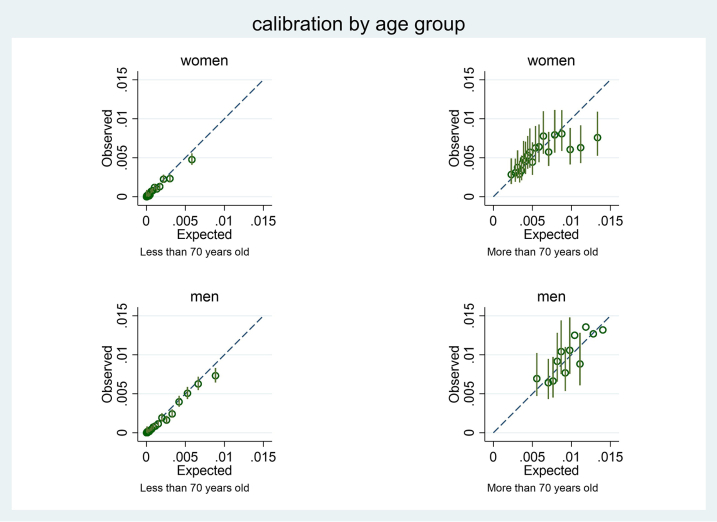


**Supplementary Figure S9:**
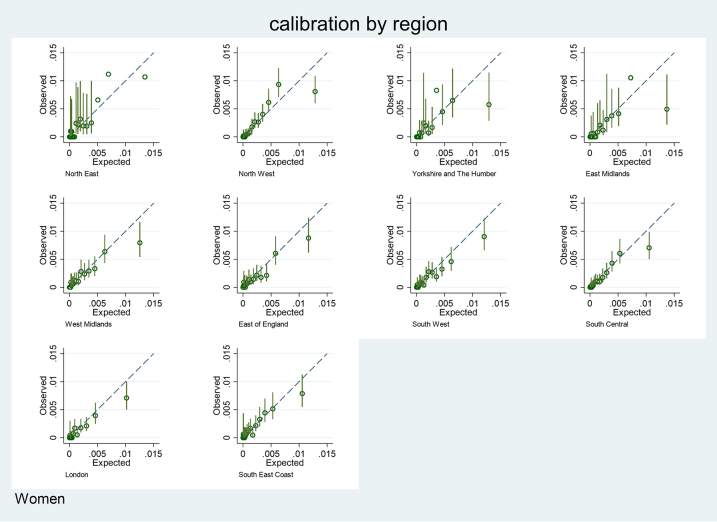


**Supplementary Figure S10:**
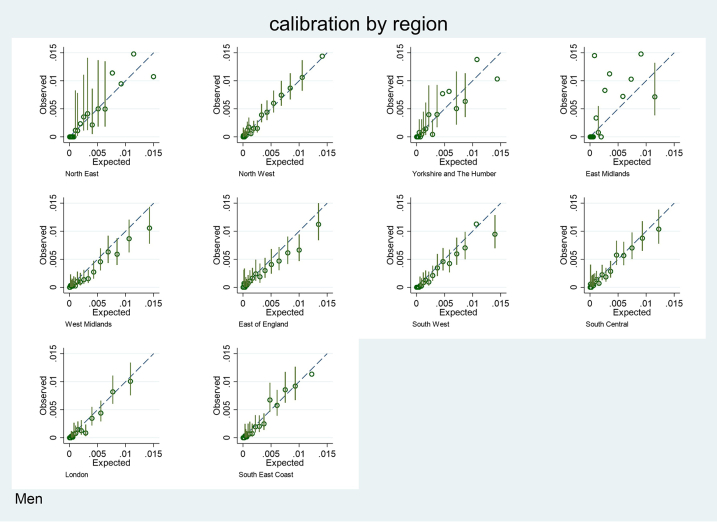


**Supplementary Figure S11:**
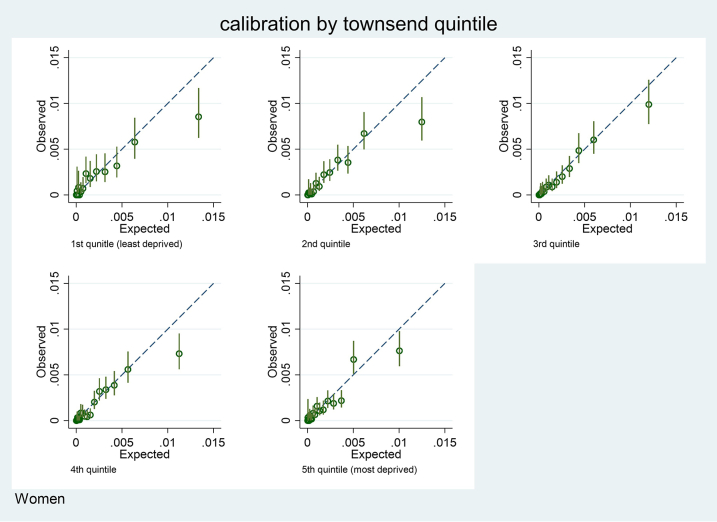


**Supplementary Figure S12:**
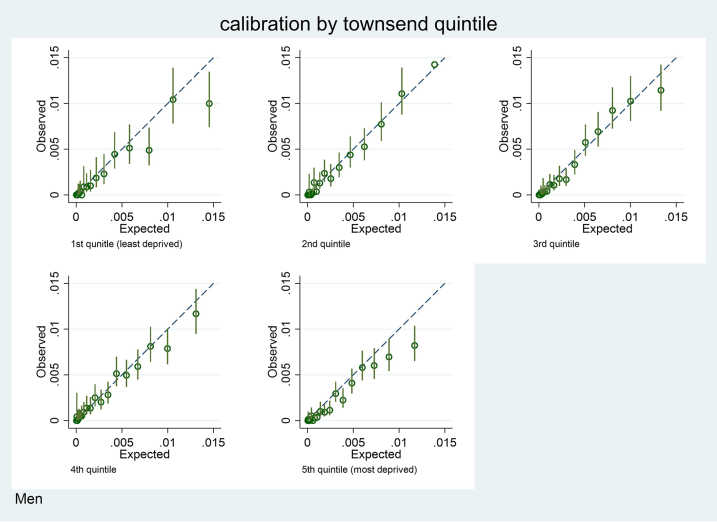


**Supplementary Figure S13:**
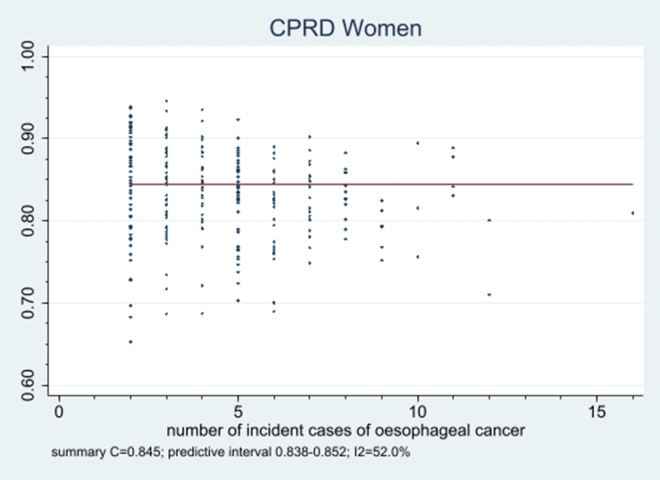


**Supplementary Figure S14:**
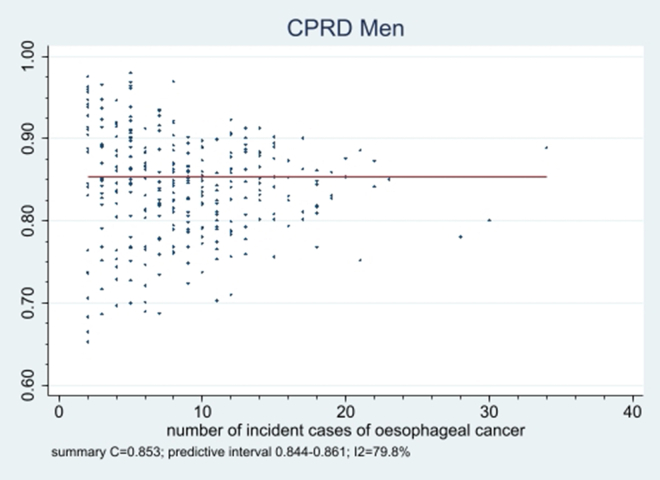


**Supplementary Figure S15:**
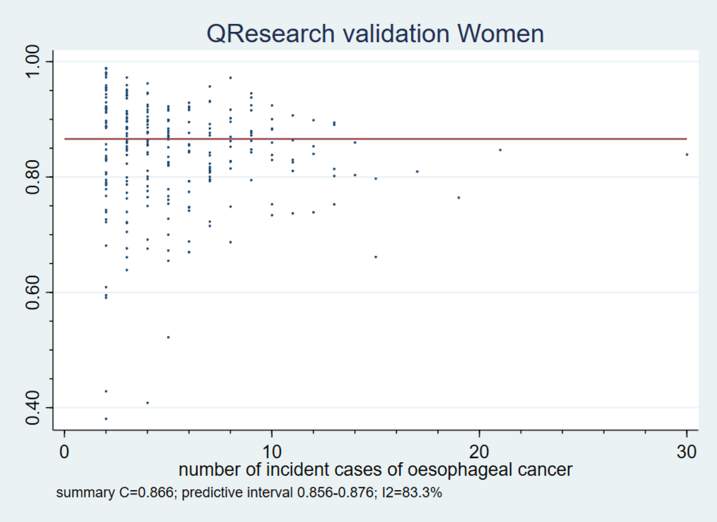


**Supplementary Figure S16:**
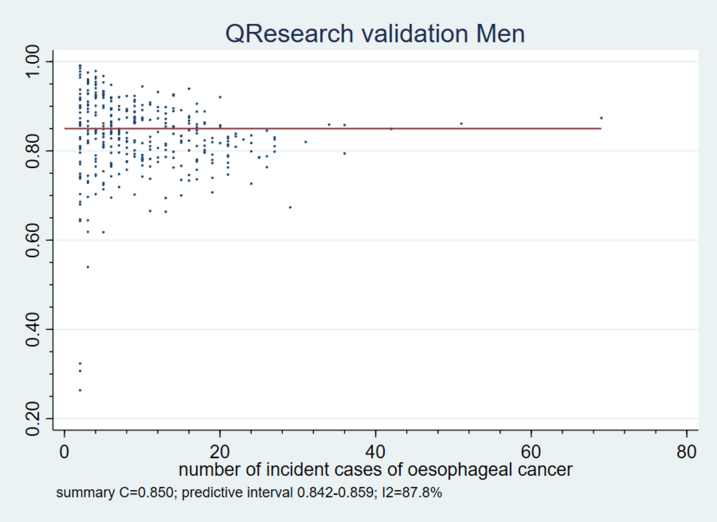


Supplementary Figure S7 shows the corresponding results for men and women in CPRD. Supplementary Figures S8–S12 show calibration by age, region and deprivation quintile for men and women. There was insufficient data to report calibration by ethnic group. Supplementary Figures S13 and S14 show the calibration measures that are meta-analysed across GP practices in the CPRD validation cohort. Supplementary Figures S15 and S16 show the corresponding results for QResearch. The results show overall the algorithms are also well calibrated in an external dataset.

### Thresholds

Table 7 shows the classification statistics of each risk algorithm in the QResearch validation cohort for men and women by twentieths of predicted risk of oesophageal cancer. For example, for the 20% of the cohort at highest predicted risk (i.e. those with a 10-year predicted risk score of 0.31% or higher), the sensitivity was 76.0%, specificity was 80.1% and the observed risk at 10 years was 0.76%. The corresponding figures for the top 30% at highest risk were a sensitivity of 88.93%, specificity of 70.1% and observed 10-year risk of 0.57%.

Supplementary Table S7 shows a comparison of classification statistics between the new risk algorithm and alternative models, evaluated for prediction of 5-year risk in the QResearch validation cohort. At cut-off values that identified approximately 30% of the cohort at highest predicted risk, the sensitivity and specificity were similar between the CanPredict algorithm (sensitivity: 57.7%, specificity: 70.1%) and Kunzmann score (sensitivity: 59.9%, specificity: 66.4%). Using cut-off values that identified approximately 20% of the cohort, the specificity of the Wang and new risk algorithm were similar (specificity 80.5% in Wang and 80.1% in CanPredict algorithm but the sensitivity was much lower in Wang (30.5%) compared with 54.5% in new risk algorithm.

### Decision curve analysis

**Supplementary Figure S17:**
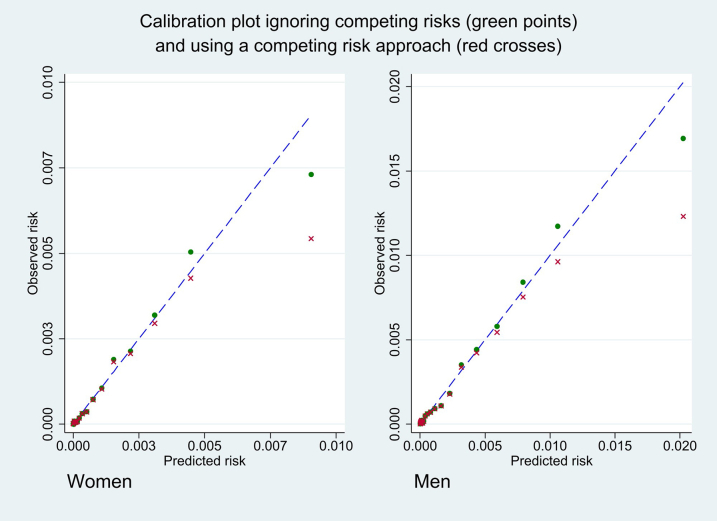


Supplementary Figures S17 and S18 show the results of the decision curve analysis for men and women for the CanPredict algorithm compared with the Kunzmann and Wang scores using the CPRD data. Overall the risk thresholds selected are low which implies that the harm of intervention is low in comparison with the benefits of correctly identifying those at highest risk. For example, Supplementary Figure S18 is presented for risk thresholds of <0.02, where a threshold of 0.02 suggests that 49 false positives would be accepted for every one true positive. It shows that the CanPredict algorithm has the highest net benefit over a range of risk threshold values below 0.02, although the absolute values are small. For example in men the net benefit is 0.0005 (5 per 10,000) at a risk threshold of 0.005 using the CanPredict algorithm.

## Discussion

To our knowledge, this study reports the first validated algorithm to predict 10-year risk of all types of oesophageal cancer using primary care electronic health records. We have included predictor variables which are accessible in everyday practice in order that the algorithm can be applied in clinical settings to identify high risk patients for further screening. The algorithms for men and women has good face validity since they included terms for age, BMI, Townsend deprivation score, smoking, alcohol, ethnicity, Barrett's oesophagus, hiatus hernia, *H. pylori* infection, use of proton pump inhibitors, anaemia, lung and blood cancer (with breast cancer in women). It is also the only algorithm to-date that was developed with an aim to capture any oesophageal cancer irrespective of its histology type. The algorithm can be applied to eligible adults aged 25–84 years in a primary care setting outside of the UK subject to local validation. The algorithm is well calibrated and has high levels of discrimination both on the QResearch validation cohort and the external CPRD validation cohort. We have provided information on sensitivity and specificity at a range of risk thresholds to inform cost-effectiveness analyses and guideline development. For example, the top 25% of patients at highest risk would capture 76% of oesophageal cancers that would develop over the next 10 years.

### Comparisons with the literature

Whilst the purpose of our algorithm differs from other studies, our associations were similar in direction to those reported elsewhere.^9^, ^10^, ^11^ For example, we found both former and current smoking are associated with an increased risk of oesophageal cancer irrespective of the histology.^9^, ^10^, ^11^ Consistent with previous studies, we also found previous or current oesophageal conditions and acid suppressant use to be associated with an increased risk of oesophageal adenocarcinoma.^9^^,^^11^ Additionally, we found prior diagnoses of lung and blood cancer in men and women and prior breast cancer in women, and prior anaemia, are positively associated with oesophageal cancer risk and the association remained significant after restricting to oesophageal adenocarcinoma (Supplementary Figures S3 and S4). This increases the clinical face validity of the variables included as predictors in the final algorithms.

In contrast with a previous risk model on oesophageal adenocarcinoma developed from hospital records,^9^ we found an association between alcohol consumption and oesophageal cancer, although the effect was diminished when we restricted the outcome of interest to oesophageal adenocarcinoma (Supplementary Figures S3 and S4). This is consistent with previous epidemiological studies of oesophageal cancer, in which alcohol drinking was not a risk factor for oesophageal adenocarcinoma but was for squamous cell cancer.^29^ We also found previous *H. pylori* infection to be associated with a lower risk of oesophageal cancer, with a stronger protective effect in oesophageal adenocarcinoma than overall cancer cases (Supplementary Figures S3 and S4) which is also consistent with previous findings.^30^, ^31^, ^32^

### Strengths and limitations

The methods to derive and validate these models are similar to those used for other clinical risk prediction tools derived from the QResearch database.^15^ Key strengths include cohort size, duration of follow up, representativeness and lack of selection, recall and respondent bias. The inclusion of more granular information on predictors is a strength in that the predictions for individual patients are likely to better reflect their individual risk although this needs to be balanced against the increased complexity of the algorithm with regards to its implementation. However, this is mitigated in settings where electronic health records are available since the majority of relevant information is already available at the point of care. There was no evidence of over-fitting. We have used a database which is regularly updated and hence the algorithm can be recalibrated and improved on a regular basis as required. This helps ensure that the algorithms will benefit from improvements in the scope and quality of the underlying database which will occur over time. This is an important strength of using routine databases for the development of risk prediction algorithms that is not feasible with prospective study cohorts that are assembled at one point in time. Our study has good face validity since it has been conducted in a community setting where the majority of patients are assessed, treated and followed up. Our database has linked hospital, mortality and cancer records^33^ for nearly all patients and is likely to have picked up nearly all cancer diagnoses thereby minimising ascertainment bias. Our validation has been done on a separate set of practices from those used to develop the algorithm although the QResearch practices all use the same GP clinical computer system (EMIS—the computer system used by 55% of UK GPs). We have also conducted an external validation using primary care data collected from a different computer system (Vision) which is a more stringent test although such studies of other risk algorithms have demonstrated comparable performance.^34^ Whilst we have undertaken an external validation, we note that this is still within the UK and hence there would be benefit from an external validation in different countries where the underlying distribution of risk factors or sub-types of oesophageal cancer may be different.

Limitations of our study include the lack of formal adjudication of cancer diagnoses and limited information on histological type as this was unspecified in 34% of cases. The algorithms also haven't taken account of previous cancer treatments or history of endoscopy due to data availability. There is potential for bias due to missing data for ethnicity, smoking, alcohol and body mass index since the data used for the analysis are collected through the course of routine medical care. However we have used multiple imputation with chained equations to address this. Our model didn't account for competing risk of non-oesophageal cancer death. This is likely to explain a degree of over-prediction of risk especially amongst the highest risk patients. However, given that the main purpose of the algorithm is to support screening then a degree of over-prediction is unlikely to be of clinical concern especially given that the predicted risks are low. Although there are developments in identifying inherited genetic predisposition to oesophageal cancer^14^^,^^35^^,^^36^ we haven't included genetic information since this is not currently routinely recorded in electronic health records and so it cannot therefore be used either to derive or validate a new prediction model or to implement it into clinical practice. Our validation cohort from CPRD did not include linked cancer registry data as this was not available within the timeframe of the study. This is likely to have resulted in a degree of under-ascertainment of cases of oesophageal cancer on the CPRD validation dataset which was more marked in women than men.

The algorithms have been designed to work in a primary care setting, making use of information which is already recorded on the GP clinical computer system. The algorithms can be integrated into the clinical computer system alongside similar algorithms which already quantify absolute risks of developing other clinical conditions including cardiovascular disease.^15^ They can be used in “batch process” mode to generate a list of patients at high risk of oesophageal cancer who may be suitable for targeted screening using Cytosponge or regular endoscopy. Alternatively they could be used within the consultation to provide information for patients on their levels of risk and screening activities.

### Conclusion

We have developed and externally validated a novel prediction algorithm which quantifies the absolute risks of oesophageal cancer in men and women. Following cost-effectiveness assessments, the algorithms could be integrated into national clinical computer systems and used to identify high risk patients for targeted screening.

## Contributors

JHC obtained funding, developed the research question and study design, defined the dataset, undertook the data manipulation, model development, primary data analysis and wrote the first draft of the paper. CC contributed to the study design, model development and validation, interpretation and drafting of the paper. XWM contributed to the study design, undertook the validation and descriptive analysis, contributed to the interpretation of results and drafting of the manuscript. RF obtained funding, contributed to the development of the research question, study protocol interpretation and drafting of the paper. JHC, CC and XWM all directly accessed the data reported in the manuscript. JHC and CC had full access to all data in the study and takes responsibility for the integrity of the data and the accuracy of the data analysis. JHC is the guarantor for the study and affirms that the manuscript is an honest, accurate, and transparent account of the study reported; that no important aspects of the study have been omitted; and that any discrepancies from the study as planned have been explained. All authors shared the decision to submit.

## Data sharing statement

To guarantee the confidentiality of personal and health information only the authors have had access to the data during the study in accordance with the relevant licence agreements. Access to the QResearch data is according to the information on the QResearch website. The full model coefficients will be published as open source software at www.canpredict.org. Data were obtained via a Clinical Practice Research Datalink (CPRD) institutional licence. Requests for data sharing should be made directly to the CPRD.

## Ethics approval

The project was reviewed in accordance with the QResearch® agreement with Derby Research Ethics Committee [reference OX39; 18/EM/0400].

## Declaration of interests

All authors have completed the ICMJE uniform disclosure form at www.icmje.org/coi_disclosure.pdf and declare: JHC is an NIHR senior investigator and reports grants from National Institute for Health Research (NIHR) Biomedical Research Centre, Oxford, grants from John Fell Oxford University Press Research Fund, grants from Cancer Research UK (CR-UK) grant number C5255/A18085, through the Cancer Research UK Oxford Centre, grants from the Oxford Wellcome Institutional Strategic Support Fund (204826/Z/16/Z) and other research councils, during the conduct of the study. JHC is an unpaid director of QResearch, a not-for-profit organisation which is a partnership between the University of Oxford and EMIS Health who supply the QResearch database used for this work. JHC has a 50% shareholding in ClinRisk Ltd, co-owning it with her husband, who is a director. As a shareholder and spouse of a director she has a financial and family interest in the ongoing and future success of the company. The company licences software both to the private sector and to NHS bodies or bodies that provide services to the NHS (through GP electronic health record providers, pharmacies, hospital providers and other NHS providers). This software implements algorithms (including similar cancer risk algorithms) developed from access to the QResearch database during her time at the University of Nottingham. CC reports grants from INNOVATE UK (to fund this work), grants from NIHR and research councils to fund other research projects outside the scope of this work and previous consultancy with ClinRisk outside the scope of the current work. RCF holds patents related to the Cytosponge and related assays licensed by the Medical Research Council to Covidien (now Medtronic). RCF reports grants from INNOVATE UK (to fund this work). RCF is named on patents related to Cytosponge and related assays which have been licensed by the Medical Research Council to Covidien GI Solutions (now Medtronic); payments to institution for webinars for Medtronic (2021), Olympus (2021) and Riche (2020); patents for Cytosponge and related assays, payment to institution for participation in Medtronic Cytosponge Advisory Board (2021) and is a co-founder and shareholder for Cyted Ltd. XWM has no interests to declare.

## Appendix A Supplementary data

Caption for supplementary material
