# Supplementary material for: Development and validation of a novel risk prediction algorithm to estimate 10-year risk of oesophageal cancer in primary care: prospective cohort study and evaluation of performance against two other risk prediction models
Source: Lancet Reg Health Eur. 2023 Aug 14;32:100700. doi: 10.1016/j.lanepe.2023.100700 (PMC10450987; doi:10.1016/j.lanepe.2023.100700)
Supplement: Caption for supplementary material [file mmc1.docx]

**Figures**

Figure 1 shows the adjusted hazard ratio (95%) for oesophageal cancer in men.

Figure 2 shows the adjusted hazard ratio (95%) for oesophageal cancer in women.

Figure 3 shows the mean predicted risks and observed risks at 10 years for men and women in the QResearch validation cohort.

Figure 4 shows the calibration plot of the model in the QResearch validation cohort.

**Supplementary Figures**

Supplementary figure 1: Adjusted hazard ratios for the fractional polynomial terms for age for oesophageal cancer.

Supplementary figure 2 Adjusted hazard ratios for the fractional polynomial terms for body mass index for oesophageal cancer.

Supplementary figure 3 Adjusted hazard ratio (95%) for oesophageal adenocarcinoma in men.

Supplementary figure 4 Adjusted hazard ratio (95%) for oesophageal adenocarcinoma in women.

Supplementary figure 5 Adjusted hazard ratio (95%) for oesophageal squamous cell carcinoma in men.

Supplementary figure 6 Adjusted hazard ratio (95%) for oesophageal squamous cell carcinoma in women.

Supplementary figure 7 Calibration plot of the model in the external validation cohort CPRD data for men and women.

Supplementary figure 8 Calibration plot of the model in the external validation cohort CPRD data for men and women by age group (under 70 and 70+).

Supplementary figure 9 Calibration plot of the model in the external validation cohort CPRD data by region in men.

Supplementary figure 10 Calibration plot of the model in the external validation cohort CPRD data by region in women .

Supplementary figure 11 Calibration plot of the model in the external validation cohort CPRD data by quintile of deprivation in men.

Supplementary figure 12 Calibration plot of the model in the external validation cohort CPRD data by quintile of deprivation in women.

Supplementary figure 13 Inter-practice variation in Harrell’s C using CPRD in women.

Supplementary figure 14 Inter-practice variation in Harrell’s C using CPRD in men.

Supplementary figure 15 Inter-practice variation in Harrell’s C using QResearch in women.

Supplementary figure 16 Inter-practice variation in Harrell’s C using QResearch in men.

Supplementary figure 17 Decision curve analysis for women in CPRD.

Supplementary figure 18 Decision curve analysis for men in CPRD.

Supplementary figure 19 Calibration plot comparing predicted and observed risk of oesophageal cancer ignoring competing risks and using a competing risk approach.
