# Supplementary material for: Development and validation of a novel risk prediction algorithm to estimate 10-year risk of oesophageal cancer in primary care: prospective cohort study and evaluation of performance against two other risk prediction models
Source: Lancet Reg Health Eur. 2023 Aug 14;32:100700. doi: 10.1016/j.lanepe.2023.100700 (PMC10450987; doi:10.1016/j.lanepe.2023.100700)
Supplement: Supplementary Tables [file mmc2.docx]

**SUPPLEMENTARY TABLES**

**Supplementary table 1a: ICD-10 and SNOMED-CT codes used to identify cases of oesophageal cancer from GP, hospital, mortality and cancer registry records.** **Details of codes for predictor variables can be found here** <https://www.qresearch.org/qcode-group-library/>

| **ICD-10 codes (group 211)** | |
| --- | --- |
| C15 | C15 - Malignant neoplasm of oesophagus |
| C150 | C150 - Malignant neoplasm: Cervical part of oesophagus |
| C151 | C151 - Malignant neoplasm: Thoracic part of oesophagus |
| C152 | C152 - Malignant neoplasm: Abdominal part of oesophagus |
| C153 | C153 - Malignant neoplasm: Upper third of oesophagus |
| C154 | C154 - Malignant neoplasm: Middle third of oesophagus |
| C155 | C155 - Malignant neoplasm: Lower third of oesophagus |
| C158 | C158 - Malignant neoplasm: Overlapping lesion of oesophagus |
| C159 | C159 - Malignant neoplasm: Oesophagus, unspecified |
|  |  |
| **SNOMED-CT codes (group 214)** | |
| 109835005 | Overlapping malignant neoplasm of esophagus (disorder) |
| 187722004 | Malignant tumour of cervical part of esophagus (disorder) |
| 187723009 | Malignant tumour of thoracic part of esophagus (disorder) |
| 187724003 | Malignant tumour of abdominal part of esophagus (disorder) |
| 187725002 | Malignant tumour of upper third of esophagus (disorder) |
| 187726001 | Malignant tumour of middle third of esophagus (disorder) |
| 187727005 | Malignant tumour of lower third of esophagus (disorder) |
| 363402007 | Malignant tumour of esophagus (disorder) |

**Supplementary table 1b: classification of oesophageal adenocarcinoma (OAC) and oesophageal squamous cell carcinoma (OSCC) based on combination of histological codes and ICD-10 codes for location derived from cancer registry**

| **Histology code (ICD-O)** | **Histology description** | **Categorisation** |
| --- | --- | --- |
| 8140 | Adenocarcinoma, NOS | OAC |
| 8141 | Small cell carcinoma, NOS | OAC |
| 8143 | Small cell carcinoma, fusiform cell | OAC |
| 8144 | Adenocarcinoma, intestinal type | OAC |
| 8145 | Adenocarcinoma, diffuse type | OAC |
| 8190 | Trabecular adenocarcinoma | OAC |
| 8200 | Adenoid cystic carcinoma | OAC |
| 8210 | Adenocarcinoma in adenomatous polyp | OAC |
| 8211 | Tubular adenocarcinoma | OAC |
| 8231 | Carcinoma simplex | OAC |
| 8260 | Papillary adenocarcinoma, NOS | OAC |
| 8261 | Adenocarcinoma in villous adenoma | OAC |
| 8262 | Villous adenocarcinoma | OAC |
| 8263 | Adenocarcinoma in tubulovillous adenoma | OAC |
| 8310 | Clear cell adenocarcinoma, NOS | OAC |
| 8401 | Apocrine adenocarcinoma | OAC |
| 8480 | Mucinous adenocarcinoma | OAC |
| 8481 | Mucin-producing adenocarcinoma | OAC |
| 8482 | Mucinous carcinoma, gastric type | OAC |
| 8490 | Signet ring cell carcinoma | OAC |
| 8550 | Acinar cell carcinoma | OAC |
| 8551 | Acinar cell cystadenocarcinoma | OAC |
| 8570 | Adenocarcinoma with squamous metaplasia | OAC |
| 8571 | Adenocarcinoma with cartilaginous and osseous metaplasia | OAC |
| 8572 | Adenocarcinoma with spindle cell metaplasia | OAC |
| 8573 | Adenocarcinoma with apocrine metaplasia | OAC |
| 8574 | Adenocarcinoma with neuroendocrine differentiation | OAC |
| 8576 | Metaplastic carcinoma, NOS | OAC |
|  |  |  |
| 8050 | Papillary carcinoma, NOS | OSCC |
| 8051 | Verrucous carcinoma, NOS | OSCC |
| 8052 | Papillary squamous cell carcinoma | OSCC |
| 8054 | Warty carcinoma | OSCC |
| 8070 | Squamous cell carcinoma, NOS | OSCC |
| 8071 | Squamous cell carcinoma, keratinizing, NOS | OSCC |
| 8072 | Squamous cell carcinoma, large cell, nonkeratinizing, NOS | OSCC |
| 8073 | Squamous cell carcinoma, small cell, nonkeratinizing | OSCC |
| 8074 | Squamous cell carcinoma, spindle cell | OSCC |
| 8075 | Squamous cell carcinoma, adenoid | OSCC |
| 8076 | Squamous cell carcinoma, microinvasive | OSCC |
| 8078 | Squamous cell carcinoma with horn formation | OSCC |
| 8083 | Basaloid squamous cell carcinoma | OSCC |
|  |  |  |
| **ICD10** | **Code description** | **categorisation** |
| C152 | Malignant neoplasm: Abdominal part of oesophagus | OAC |
| C155 | Malignant neoplasm: Lower third of oesophagus | OAC |
| C158 | Malignant neoplasm: Overlapping lesion of oesophagus | OAC |
| C159 | Malignant neoplasm: Oesophagus, unspecified | OAC |
| C150 | Malignant neoplasm: Cervical part of oesophagus | OSCC |
| C151 | Malignant neoplasm: Thoracic part of oesophagus | OSCC |
| C153 | Malignant neoplasm: Upper third of oesophagus | OSCC |
| C154 | Malignant neoplasm: Middle third of oesophagus | OSCC |
|  |  |  |

**Supplementary table 2 Alternative risk models**

|  | **Kunzmann AT (2018)^1^** | **Wang QL (2021)^2^** |
| --- | --- | --- |
| Outcomes | Oesophageal adenocarcinoma | Oesophageal squamous cell carcinoma |
| Model Type | Prognostic model converted into a points based scoring system | Competing risk regression model |
| Study design | Cohort but used logistic regression | Cohort with competing risk regression |
| Setting summary | Community (UK Biobank) | Community |
| Sex | Both | Both |
| Age range | 50+ | 40+ |
| Country (s) | UK | Norway |
| Sample size total | 355,034 | Derivation: 77,476  Validation: 3,033,715 |
| Sample size cancer cases | 220 | Derivation: 53  Validation: 105 |
| Number of years of follow up | 5 | Up to 15 |
| outcome details | Histologically confirmed | Histologically confirmed |
| Model parameters | Age (50-54; 55-59; 60-64; 65+) | Age (40-49, 50-59, 60+) |
|  | Sex | Sex |
|  | BMI (<25; 25-29.9; 30-34.9; 35+) | BMI (<25, 25+) |
|  | Smoking (current/former/never) | Smoking (current/former/never) |
|  | Alcohol not included | Alcohol (<3 times/week 3+ times/week) |
|  | Reflux medication (none vs any, as one of the oesophageal conditions below) | Medication not included |
|  | Oesophageal conditions including any of the following as a combined variable: self-reported history of gastroesophageal reflux disease, Barrett’s oesophagus, hiatus hernia, or oesophageal stricture and/ or oesophageal fundoplication or hiatus hernia surgery and/or anti-reflux medication use (none or any). | None included |
|  | Hiatus hernia (under the umbrella term of oesophageal conditions) | No hiatus hernia |
| Validation | internal | Internal and external |
| **Performance metrics** |  |  |
| C or AUROC statistic | 0.80 (0.78 to 0.82) | 5-year risk:  0.76 (0.58-0.93) on derivation; 0.67(0.45-0.89) on internal validation; and 0.70 (0.64-0.75) on external validation  10-year risk:  0.74 (0.61-0.88) on derivation and 0.68 (0.51-0.85) on internal validation (no external)  15-year risk:  0.77(0.66-0.87) on derivation and 0.73(0.62-0.85) on internal validation (no external validation) |
| Royston’s D statistic | Not reported | 5-year: 0.51 on derivation; 0.35 on internal validation; and 0.39 on external validation  10-year: 0.49 on derivation and 0.36 on internal validation (no external)  15-year: 0.53 on derivation and 0.46 on internal validation (no external) |
| R^2^ explained variation | Not reported | Not reported |
| calibration | Well calibrated. | Well calibrated: external calibration: a = -0.121 and calibration slope (b)= 1.110 |
| Sensitivity/specificity at a threshold | A threshold (8+ points) identified 29.5% of the population with 77.4 sensitivity and 70.5% specificity | Score of 49 or over has sensitivity of 56.8% and specificity of 74% |
| limitations | Self-reported medical history and medication use; no information on degree and duration of medication use for reflux; healthy participant effect of UK Biobank | Limited number of predictors; no anti-reflex medication or any other medication use; no comorbidities recorded; model performance suboptimal |

**Supplementary table 3a: Flow of patients through the study in the QResearch derivation validation cohort and the CPRD validation cohort.**

|  | **QResearch Derivation**  **cohort** | **QResearch Validation**  **cohort** | **CPRD validation cohort** |
| --- | --- | --- | --- |
| Eligible GP practices | 1,354 | 450 | 363 |
| Aged 25-84 years | 13,053,500 | 4,155,792 | 3,294,988 |
| Missing Townsend score | 46,256 | 13,290 | 749,944 |
|  |  |  |  |
| **Conditions prior to or at cohort entry** |  |  |  |
| Oesophageal cancer | 6,063 | 1,931 | 668 |
| Oesophageal operations | 125 | 29 | 0 |
| Gastric cancer | 3,535 | 1,156 | 454 |
| Prior oral cancer | 10,724 | 3,469 | 1194 |
| Prior oesophageal varices | 6,030 | 1,874 | 918 |
| Liver cirrhosis | 41,418 | 13,015 | 7463 |
|  |  |  |  |
| **New onset of alarm symptoms in the 90 days prior to cohort entry** |  |  |  |
| New onset gastrointestinal bleeding* | 2,502 | 774 | 674 |
| New onset weight loss* | 5,483 | 1,718 | 1,717 |
| New onset dysphagia* | 3,212 | 1,009 | 999 |
|  |  |  |  |
| Target population after exclusions | 12,928,152 (99.0) | 4,117,527 (99.1) | 2,531,700 (76.8) |
|  |  |  |  |
| **Outcomes during follow-up - values are numbers (%)** |  |  |  |
| incident cases of oesophageal cancer | 16,384 (0.13) | 5,014 (0.12) | 3930 (0.16) |
| Men | 11,535 (70.4) | 3,529 (70.4) | 2703 (68.8) |
| Women | 4,849 (29.6) | 1,485 (29.6) | 1227 (31.2) |
| Total person years of follow up | 74,901,724 | 23,725,161 | 15,946,841 |
| Median (IQR) years | 5.8 (1.8 to 8.5) | 5.8 (1.7 to 8.5) | 6.3 (2.9 to 10.4) |
| crude rates per 10,000 pyrs (95% CI) | 2.19 (2.15 to 2.22) | 2.11 (2.06 to 2.17) | 2.46 (2.39 to 2.54) |

**Supplementary table 3b Age-standardised incidence rate of oesophageal cancer per 10,000 person-years by ethnic groups and by gender in the QResearch derivation cohort**

| **Ethnic groups** | **Overall (95% CI)** | **Female (95% CI)** | **Male (95% CI)** |
| --- | --- | --- | --- |
| White | 4.63 (4.55 to 4.7) | 2.63 (2.55 to 2.71) | 6.70 (6.57 to 6.83) |
| Indian | 1.18 (0.96 to 1.4) | 0.74 (0.49 to 1.00) | 1.62 (1.26 to 1.97) |
| Pakistani | 0.69 (0.48 to 0.91) | 0.60 (0.30 to 0.89) | 0.77 (0.46 to 1.07) |
| Bangladeshi | 1.55 (1.17 to 1.93) | 1.88 (1.29 to 2.48) | 1.19 (0.73 to 1.66) |
| Other Asian | 0.97 (0.69 to 1.25) | 0.66 (0.3 to 1.02) | 1.30 (0.87 to 1.74) |
| Caribbean | 1.64 (1.37 to 1.90) | 1.09 (0.79 to 1.38) | 2.27 (1.81 to 2.73) |
| Black African | 0.44 (0.32 to 0.57) | 0.24 (0.12 to 0.37) | 0.69 (0.45 to 0.93) |
| Chinese | 0.78 (0.35 to 1.20) | 0.26 (0.01 to 0.53) | 1.30 (0.51 to 2.10) |
| other | 1.01 (0.8 to 1.22) | 0.79 (0.51 to 1.08) | 1.28 (0.95 to 1.61) |
| Missing ethnicity | 4.92 (4.8 to 5.03) | 3.1 (2.97 to 3.24) | 6.52 (6.33 to 6.7) |

**Supplementary Table 3c: crude incidence of oesophageal cancer per 10,000 person years in the CPRD validation cohort identified on combinations of three different data sources.**

|  | **Cases Identified on GP record** | **Incidence rate on GP record** | **Cases identified on either GP or ONS** | **Incidence rate on GP or ONS record** | **Cases Identified on GP or ONS or HES** | **Incidence rate on either GP or ONS or HES** |
| --- | --- | --- | --- | --- | --- | --- |
|  |  |  |  |  |  |  |
| <34 years | 5 | 0.03 (0.01 to 0.08) | 5 | 0.03 (0.01 to 0.08) | 6 | 0.04 (0.02 to 0.09) |
| 35-39 years | 32 | 0.16 (0.11 to 0.22) | 32 | 0.16 (0.11 to 0.22) | 37 | 0.18 (0.13 to 0.25) |
| 40-44 years | 74 | 0.36 (0.29 to 0.46) | 77 | 0.38 (0.30 to 0.47) | 88 | 0.43 (0.35 to 0.53) |
| 45-49 years | 138 | 0.78 (0.66 to 0.92) | 145 | 0.81 (0.69 to 0.96) | 168 | 0.94 (0.81 to 1.10) |
| 50-54 years | 271 | 1.69 (1.50 to 1.90) | 280 | 1.74 (1.55 to 1.96) | 323 | 2.01 (1.80 to 2.24) |
| 55-59 years | 431 | 2.59 (2.36 to 2.85) | 450 | 2.70 (2.46 to 2.96) | 506 | 3.04 (2.79 to 3.32) |
| 60-64 years | 487 | 3.69 (3.38 to 4.03) | 508 | 3.85 (3.53 to 4.20) | 566 | 4.29 (3.95 to 4.66) |
| 65-69 years | 497 | 4.55 (4.17 to 4.97) | 522 | 4.78 (4.38 to 5.20) | 600 | 5.49 (5.07 to 5.95) |
| 70-74 years | 542 | 6.00 (5.52 to 6.53) | 582 | 6.45 (5.94 to 6.99) | 661 | 7.32 (6.79 to 7.90) |
| 75-79 years | 472 | 6.89 (6.30 to 7.54) | 511 | 7.46 (6.84 to 8.14) | 592 | 8.65 (7.98 to 9.37) |
| 80-84 years | 282 | 7.29 (6.49 to 8.20) | 320 | 8.28 (7.42 to 9.23) | 383 | 9.91 (8.96 to 10.95) |
|  |  |  |  |  |  |  |
| female | 1010 | 1.24 (1.16 to 1.32) | 1065 | 1.31 (1.23 to 1.39) | 1227 | 1.51 (1.42 to 1.59) |
| male | 2221 | 2.85 (2.73 to 2.97) | 2367 | 3.04 (2.92 to 3.16) | 2703 | 3.47 (3.34 to 3.60) |
|  |  |  |  |  |  |  |
| **total** | **3231** | **2.03 (1.96 to 2.10)** | **3432** | **2.15 (2.08 to 2.23)** | **3930** | **2.46 (2.39 to 2.54)** |

**Table 3d** Crude incidence rates (95%CI) by calendar year for the QResearch derivation cohort and CPRD validation cohort. QResearch cases are identified from either GP, HES, ONS or cancer registry records. CPRD rates are identified from GP or HES or ONS records.

|  | QResearch |  |  | CPRD |  |  |
| --- | --- | --- | --- | --- | --- | --- |
|  | cases | pyrs | Rate per 10,000 | cases_4 | pyrs_4 | Rate per 10,000 |
| total | 16384 | 74896504 | 2.19 (2.15 to 2.22) | 3930 | 15946841 | 2.46 (2.39 to 2.54) |
| 2005 | 796 | 4335232 | 1.84 (1.71 to 1.97) | 313 | 1478362 | 2.12 (1.90 to 2.37) |
| 2006 | 844 | 4357574 | 1.94 (1.81 to 2.07) | 377 | 1518760 | 2.48 (2.24 to 2.75) |
| 2007 | 917 | 4429875 | 2.07 (1.94 to 2.21) | 358 | 1550148 | 2.31 (2.08 to 2.56) |
| 2008 | 974 | 4508401 | 2.16 (2.03 to 2.30) | 382 | 1582454 | 2.41 (2.18 to 2.67) |
| 2009 | 980 | 4620332 | 2.12 (1.99 to 2.26) | 393 | 1590302 | 2.47 (2.24 to 2.73) |
| 2010 | 1142 | 4791457 | 2.38 (2.25 to 2.53) | 399 | 1597556 | 2.50 (2.26 to 2.76) |
| 2011 | 1117 | 4957060 | 2.25 (2.13 to 2.39) | 398 | 1560358 | 2.55 (2.31 to 2.81) |
| 2012 | 1238 | 5107251 | 2.42 (2.29 to 2.56) | 379 | 1492921 | 2.54 (2.30 to 2.81) |
| 2013 | 1204 | 4966355 | 2.42 (2.29 to 2.57) | 367 | 1413338 | 2.60 (2.34 to 2.88) |
| 2014 | 1085 | 4853147 | 2.24 (2.11 to 2.37) | 325 | 1216500 | 2.67 (2.40 to 2.98) |
| 2015 | 1111 | 4976308 | 2.23 (2.11 to 2.37) | 239 | 946078 | 2.53 (2.23 to 2.87) |
| 2016 | 1182 | 5214511 | 2.27 (2.14 to 2.40) | n/a | n/a | n/a |
| 2017 | 1161 | 5321284 | 2.18 (2.06 to 2.31) | n/a | n/a | n/a |
| 2018 | 1181 | 5403034 | 2.19 (2.06 to 2.31) | n/a | n/a | n/a |
| 2019 | 1149 | 5658092 | 2.03 (1.92 to 2.15) | n/a | n/a | n/a |
| 2020 | 303 | 1396597 | 2.17 (1.94 to 2.43) | n/a | n/a | n/a |

**Supplementary table 3e. Crude incidence rates (95%CI) by geographical region for the QResearch derivation cohort and CPRD validation cohort. QResearch cases are identified from either GP, HES, ONS or cancer registry records. CPRD rates are identified from GP or HES or ONS records.**

|  | QResearch |  | CPRD |  |
| --- | --- | --- | --- | --- |
|  | cases | Rates (95% CI) | cases | Rates (95% CI) |
| East Midlands | 723 | 2.59 (2.40 to 2.78) | 114 | 3.30 (2.75 to 3.97) |
| East of England | 946 | 2.18 (2.05 to 2.33) | 793 | 3.08 (2.87 to 3.30) |
| London | 2017 | 1.18 (1.13 to 1.24) | 186 | 2.72 (2.36 to 3.14) |
| North East | 642 | 2.50 (2.32 to 2.70) | 119 | 2.73 (2.28 to 3.26) |
| North West | 3610 | 2.71 (2.63 to 2.80) | 460 | 2.43 (2.22 to 2.66) |
| South Central | 2011 | 2.04 (1.96 to 2.13) | 411 | 2.18 (1.98 to 2.40) |
| South East | 1544 | 2.50 (2.38 to 2.63) | 486 | 2.51 (2.30 to 2.74) |
| South West | 1924 | 2.56 (2.45 to 2.68) | 513 | 2.35 (2.16 to 2.56) |
| West Midlands | 2017 | 2.65 (2.53 to 2.76) | 381 | 1.80 (1.63 to 1.99) |
| Yorkshire & Humber | 950 | 2.57 (2.41 to 2.74) | 467 | 2.47 (2.25 to 2.70) |
| total | 16384 | 2.19 (2.15 to 2.22) | 3930 | 2.46 (2.39 to 2.54) |

**Supplementary table 4a: Characteristics of incident cases of oesophageal cancer (identified from any of the four linked data sources) in the QResearch derivation and validation cohorts. Linked cancer registry data for histological type were not available for CPRD.**

|  | **QResearch Derivation** | **QResearch Validation** | **CPRD validation cohort** |
| --- | --- | --- | --- |
|  | **16384** | **5014** | **3930** |
| **Histological type** |  |  |  |
| Adenocarcinoma | 7561 (46.1) | 2294 (45.8) | n/a |
| Squamous carcinoma | 3548 (21.7) | 1101 (22.0) | n/a |
| Other | 640 (3.9) | 210 (4.2) | n/a |
| Not recorded | 4635 (28.3) | 1409 (28.1) | n/aSUPPP |
| Men | 11535 (70.4) | 3529 (70.4) | 2703 (68.8) |
| Mean age at diagnosis in men (SD) | 69.2 (10.8) | 69.2 (10.7) | 69.3 (10.3) |
| Mean age at diagnosis in women (SD) | 72.4 (11.0) | 72.7 (10.8) | 72.4 (11.1) |
| mean Townsend score (SD) | -0.3 (3.0) | -0.4 (3.0) | n/a |
| BMI recorded | 14740 (90.0) | 4569 (91.1) | 2792 (71.0) |
| mean BMI (SD) | 27.3 (5.2) | 27.3 (5.3) | 26.8 (4.6) |
| **Townsend quintile^a^** |  |  |  |
| 1 (most affluent) | 4622 (28.2) | 1500 (29.9) | 832 (21.2) |
| 2 | 4049 (24.7) | 1271 (25.3) | 969 (24.7) |
| 3 | 3324 (20.3) | 973 (19.4) | 872 (22.2) |
| 4 | 2632 (16.1) | 725 (14.5) | 771 (19.6) |
| 5 (most deprived) | 1757 (10.7) | 545 (10.9) | 486 (12.4) |
| **Ethnicity** |  |  |  |
| White | 10660 (65.1) | 3334 (66.5) | 1342 (34.1) |
| Indian | 74 (0.5) | 43 (0.9) | 11 (0.3) |
| Pakistani | 36 (0.2) | * | * |
| Bangladeshi | 58 (0.4) | 11 (0.2) | * |
| Other Asian | 34 (0.2) | 21 (0.4) | * |
| Caribbean | 103 (0.6) | 47 (0.9) | * |
| Black African | 45 (0.3) | 10 (0.2) | * |
| Chinese | 14 (0.1) | * | * |
| Other | 90 (0.5) | 19 (0.4) | 5 (0.1) |
| Ethnicity not recorded | 5270 (32.2) | 1521 (30.3) | 2554 (65.0) |
|  |  |  |  |
| **Smoking** |  |  |  |
| Non-smoker | 6089 (37.2) | 1925 (38.4) | 1277 (32.5) |
| Ex-smoker | 4980 (30.4) | 1518 (30.3) | 889 (22.6) |
| Light smoker | 3416 (20.8) | 992 (19.8) | 722 (18.4) |
| Moderate smoker | 671 (4.1) | 174 (3.5) | 549 (14.0) |
| Heavy smoker | 581 (3.5) | 230 (4.6) | 460 (11.7) |
| Smoking not recorded | 647 (3.9) | 175 (3.5) | 33 (0.8) |
|  |  |  |  |
| **Alcohol status** |  |  |  |
| Non-drinker | 8951 (54.6) | 2754 (54.9) | 541 (13.8) |
| Trivial <1u/day | 2324 (14.2) | 736 (14.7) | 1405 (35.8) |
| Light 1-2u/day | 1209 (7.4) | 368 (7.3) | 970 (24.7) |
| Moderate 3-6u/day | 1677 (10.2) | 534 (10.7) | 419 (10.7) |
| Heavy 7-9u/day | 259 (1.6) | 86 (1.7) | 60 (1.5) |
| Very Heavy >9u/day | 115 (0.7) | 35 (0.7) | 54 (1.4) |
| Alcohol not recorded | 1849 (11.3) | 501 (10.0) | 481 (12.2) |
|  |  |  |  |
| **Current medication** |  |  |  |
| H2 blockers | 196 (1.2) | 58 (1.2) | n/a |
| NSAID | 1393 (8.5) | 434 (8.7) | n/a |
| Statins | 562 (3.4) | 203 (4.0) | n/a |
| Aspirin | 1999 (12.2) | 623 (12.4) | n/a |
|  |  |  |  |
| **Proton pump inhibitor exposure** |  |  |  |
| None | 12041 (73.5) | 3733 (74.5) | 2940 (74.8) |
| 1-5 scripts | 1847 (11.3) | 568 (11.3) | 431 (11.0) |
| 6-11 scripts | 470 (2.9) | 143 (2.9) | 130 (3.3) |
| 12-23 scripts | 544 (3.3) | 144 (2.9) | 146 (3.7) |
| 24-47 scripts | 669 (4.1) | 203 (4.0) | 139 (3.5) |
| 48+ scripts | 813 (5.0) | 223 (4.4) | 144 (3.7) |
|  |  |  |  |
| **Medical conditions** |  |  |  |
| Type 2 diabetes | 1579 (9.6) | 501 (10.0) | n/a |
| Barrett’s oesophagus** | 602 (3.7) | 158 (3.2) | 135 (3.4) |
| Blood cancer | 120 (0.7) | 28 (0.6) | 25 (0.6) |
| Breast cancer | 180 (1.1) | 71 (1.4) | 46 (1.2) |
| Lung cancer | 43 (0.3) | 11 (0.2) | 6 (0.2) |
| Peptic ulcer disease | 961 (5.9) | 271 (5.4) | 242 (6.2) |
| Pernicious anaemia | 86 (0.5) | 24 (0.5) | n/a |
| Hiatus hernia | 1203 (7.3) | 306 (6.1) | 306 (7.8) |
| Gastro-oesophageal reflux | 1396 (8.5) | 405 (8.1) | n/a |
| Indigestion |  |  | n/a |
| H. *pylori* infection | 308 (1.9) | 88 (1.8) | 58 (1.5) |
| Family history bowel cancer | 297 (1.8) | 114 (2.3) | 33 (0.8) |

^a^ The Townsend score is an area-level score based on the patients’ postcode which includes unemployment; non-car ownership and household overcrowding evaluated for a given area of approximately 120 households. A greater Townsend score implies a greater level of deprivation.

* are cells with observations <5 counts

** Of the 7651 in the derivation cohort with OAC, 420 (5.55%) had Barrett's. Of the 3548 with OSCC, 27 (0.76%) had Barrett’s. Of the remaining 640 cases which were not identified as either OAC or OSCC, 18 (2.91%) had Barrett's oesophagus”

**Supplementary table 4b Number of incident cases of oesophageal cancer by year of follow up in QResearch and CPRD**

| **Year of follow up** | **QResearch derivation cohort** | **QResearch validation cohort** | **CPRD validation cohort** |
| --- | --- | --- | --- |
| **<1** | 902 | 280 | 217 |
| **1** | 1,725 | 549 | 417 |
| **2** | 1,473 | 484 | 448 |
| **3** | 1,504 | 431 | 385 |
| **4** | 1,393 | 459 | 440 |
| **5** | 1,312 | 399 | 402 |
| **6** | 1,176 | 371 | 352 |
| **7** | 1,110 | 341 | 358 |
| **8** | 1,120 | 339 | 301 |
| **9** | 834 | 278 | 292 |
| **10** | 791 | 255 | 238 |
| **11** | 749 | 209 | 80 |
| **12** | 656 | 203 | **n/a** |
| **13** | 689 | 183 | **n/a** |
| **14** | 561 | 145 | **n/a** |
| **15** | 389 | 88 | **n/a** |
| **Total** | **16384** | **5014** | **3930** |

**Supplementary table 5 Performance of each risk algorithm in the QResearch validation cohort by age band**

|  |  | **CanPredict algorithm**  **10 year risk overall oesophageal cancer** | **Kunzman**  **5 year risk**  **OAC** | **Wang**  **10 year risk**  **OSCC** |
| --- | --- | --- | --- | --- |
| statistic | Age band (years) | Mean (95% CI) | Mean (95% CI) | Mean (95% CI) |
|  |  |  |  |  |
| Harrell's C | 25-49 | .7 (.662 to .739) | N/A | .589 (.553 to .624) |
|  | 50-59 | .739 (.721 to .757) | .723 (.708 to .739) | .595 (.574 to .616) |
|  | 60-69 | .711 (.695 to .726) | .684 (.669 to .698) | .58 (.563 to .597) |
|  | 70-79 | .655 (.637 to .673) | .638 (.622 to .654) | .544 (.526 to .562) |
|  | 80+ | .617 (.585 to .65) | .593 (.56 to .626) | .538 (.506 to .571) |
|  |  |  |  |  |
| Royston’s D statistic | 25-49 | 1.45 (1.24 to 1.66) | N/A | .529 (.368 to .691) |
|  | 50-59 | 1.49 (1.38 to 1.61) | 1.23 (1.13 to 1.33) | .509 (.409 to .61) |
|  | 60-69 | 1.27 (1.18 to 1.36) | 1.03 (.953 to 1.12) | .415 (.333 to .497) |
|  | 70-79 | .909 (.806 to 1.01) | .762 (.676 to .848) | .218 (.125 to .31) |
|  | 80+ | .646 (.473 to .818) | .475 (.316 to .634) | * |
|  |  |  |  |  |
| R^2^ statistic | 25-49 | 33.4 (27 to 39.9) | N/A | 6.27 (2.68 to 9.86) |
|  | 50-59 | 34.8 (31.4 to 38.2) | 26.6 (23.5 to 29.6) | 5.84 (3.68 to 8) |
|  | 60-69 | 27.8 (24.9 to 30.8) | 20.4 (17.8 to 22.9) | 3.95 (2.44 to 5.46) |
|  | 70-79 | 16.5 (13.4 to 19.6) | 12.2 (9.75 to 14.6) | 1.12 (.178 to 2.07) |
|  | 80+ | 9.06 (4.67 to 13.5) | 5.11 (1.86 to 8.36) | * |

* are cells with too few observations to be estimated reliably

**Supplementary table 6 sensitivity and specificity of Kunzmann and Wang scores compared with the new risk prediction algorithm in prediction of 5-year risk in the QResearch validation cohort.**

| **Risk score** | **Cut-off** ^a^ | **% cohort above cut-off** | **Sensitivity**  **%** | **Specificity**  **%** | **Observed 5-year risk %** |
| --- | --- | --- | --- | --- | --- |
|  |  |  |  |  |  |
| Age 50-84 |  |  |  |  |  |
| CanPredict | 0.220 | top 33.7% | 62.69 | 66.39 | 0.45 (0.43, 0.47) |
| Kunzmann | Score of 8 | top 33.7% | 59.88 | 66.38 | 0.38 (0.37, 0.41) |
|  |  |  |  |  |  |
| Age 40-84 |  |  |  |  |  |
| CanPredict | 0.239 | top 19.7% | 53.73 | 80.37 | 0.47 (0.45, 0.50) |
| Wang | Score of 49 | top 19.7% | 30.45 | 80.48 | 0.23 (0.21, 0.25) |

*Men or women over the age of 55, with at least 6 prescriptions for PPI with the last prescription being within 6 months from study entry

^a^ Score cut-off in CanPredict model is determined based on Cohort restricted to individuals aged 50 and over in comparison with the Kunzmann model; and aged 40 and over in comparison with the Wang score. **Supplementary table 7a Model performance for CanPredict by ethnicity and ageband in QResearch validation cohort**

|  | **women** | **men** |
| --- | --- | --- |
|  | Mean (95CI) | Mean (95CI) |
| **Harrell’s C** |  |  |
| **Ethnic groups** |  |  |
| White | .852 (.842 to .862) | .843 (.837 to .849) |
| Indian | .908 (.847 to .97) | .888 (.83 to .945) |
| Pakistani | .722 (.398 to 1.04) | .849 (.726 to .972) |
| Bangladeshi | .944 (.905 to .983) | .768 (.563 to .974) |
| Other Asian | .83 (.726 to .935) | .852 (.775 to .93) |
| Caribbean | .887 (.843 to .932) | .779 (.714 to .843) |
| Black African | .874 (.787 to .96) | .861 (.771 to .951) |
| Chinese | .968 (.947 to .988) | .962 (.931 to .993) |
| Other | .822 (.676 to .968) | .861 (.782 to .94) |
|  |  |  |
| **Ageband** |  |  |
| 25-49 | 0.681 (.621 to .741) | 0.757 (.718 to .795) |
| 50-59 | 0.671 (.626 to .717) | 0.679 (.656 to .702) |
| 60-69 | 0.652 (.620 to .684) | 0.661 (.641 to .68) |
| 70-79 | 0.585 (.555 to .615) | 0.597 (.573 to .620) |
| 80+ | 0.561 (.513 to .610) | 0.611 (.565 to .657) |
|  |  |  |
| **Royston’s D statistic** |  |  |
| **Ethnic groups** |  |  |
| White | 2.28 (2.18 to 2.38) | 2.13 (2.07 to 2.20) |
| Indian | 2.84 (1.98 to 3.71) | 2.84 (2.27 to 3.42) |
| Pakistani | 2.95 (.696 to 5.20) | 2.30 (1.35 to 3.26) |
| Bangladeshi | 2.92 (1.22 to 4.62) | 1.86 (.552 to 3.16) |
| Other Asian | 2.52 (1.48 to 3.57) | 2.16 (1.19 to 3.13) |
| Caribbean | 2.32 (1.53 to 3.11) | 1.82 (1.18 to 2.45) |
| Black African | 2.63 (1.08 to 4.19) | 2.45 (1.11 to 3.79) |
| Chinese | 3.01 (.537 to 5.49) | 2.81 (.881 to 4.75) |
| Other | 3.11 (1.91 to 4.32) | 2.42 (1.52 to 3.31) |
|  |  |  |
| **Ageband** |  |  |
| 25-49 | 2.30 (1.92 to 2.67) | 1.93 (1.71 to 2.14) |
| 50-59 | 1.24 (.996 to 1.49) | 1.13 (1.00 to 1.26) |
| 60-69 | .969 (.782 to 1.16) | .953 (.843 to 1.06) |
| 70-79 | .524 (.361 to .686) | .596 (.462 to .731) |
| 80+ | .357 (.0974 to .616) | .572 (.329 to .815) |
|  |  |  |
| **R^2^ explained variation** |  |  |
| **Ethnic groups** |  |  |
| White | 55.4 (53.1 to 57.6) | 52.1 (50.5 to 53.6) |
| Indian | 65.9 (52.2 to 79.5) | 65.8 (56.8 to 74.9) |
| Pakistani | 66.7 (29.8 to 104) | 55.8 (35.4 to 76.1) |
| Bangladeshi | 66.8 (40.8 to 92.7) | 44.8 (9.05 to 80.6) |
| Other Asian | 60.2 (40.3 to 80.2) | 52.4 (29.6 to 75.2) |
| Caribbean | 56.2 (39.5 to 73.0) | 44.1 (26.9 to 61.3) |
| Black African | 61.7 (32.0 to 91.4) | 58.2 (31.2 to 85.1) |
| Chinese | 67.9 (33.3 to 102) | 64.6 (32.8 to 96.3) |
| Other | 69.6 (53.8 to 85.5) | 58.0 (40.1 to 75.9) |
|  |  |  |
| **Ageband** |  |  |
| 25-49 | 55.7 (47.7 to 63.8) | 47.0 (41.4 to 52.6) |
| 50-59 | 27.0 (19.1 to 34.8) | 23.5 (19.4 to 27.6) |
| 60-69 | 18.3 (12.6 to 24.1) | 17.8 (14.4 to 21.2) |
| 70-79 | 6.14 (2.56 to 9.72) | 7.83 (4.56 to 11.1) |
| 80+ | * | 7.26 (1.53 to 13) |

**Supplemental table 7b Performance of the CanPredict risk algorithm in prediction of oesophageal adenocarcinoma 10-year risk in the QResearch validation cohort**

|  |  | **women** | **men** |
| --- | --- | --- | --- |
| statistic |  | Mean (95CI) | Mean (95CI) |
| Harrell's C | **overall** | **.86 (.844 to .875)** | **.852 (.844 to .86)** |
|  | **Ethnic groups** |  |  |
|  | White | .848 (.841 to .856) | .841 (.837 to .844) |
|  | Indian | .906 (.866 to .946) | .874 (.837 to .911) |
|  | Pakistani | .966 (.94 to .992) | .821 (.754 to .888) |
|  | Bangladeshi | .996 (.995 to .996) | .819 (.733 to .905) |
|  | Other Asian | .91 (.843 to .976) | .806 (.754 to .858) |
|  | Caribbean | .919 (.882 to .955) | .861 (.823 to .899) |
|  | Black African | .966 (.943 to .989) | .857 (.804 to .91) |
|  | Chinese | .916 (.833 to 1) | .956 (.936 to .975) |
|  | Other | .731 (.588 to .874) | .821 (.752 to .891) |
|  | **Age groups** |  |  |
|  | 25-49 | .58 (.499 to .66) | .786 (.742 to .829) |
|  | 50-59 | .698 (.632 to .764) | .693 (.662 to .723) |
|  | 60-69 | .625 (.57 to .679) | .643 (.617 to .668) |
|  | 70-79 | .567 (.514 to .621) | .602 (.573 to .632) |
|  | 80+ | .539 (.461 to .616) | .607 (.544 to .669) |
|  |  |  |  |
| Royston’s D statistic | **overall** | **2.41 (2.25 to 2.58)** | **2.24 (2.16 to 2.33)** |
|  | **Ethnic groups** |  |  |
|  | White | 2.31 (2.14 to 2.48) | 2.13 (2.05 to 2.21) |
|  | Indian | 3.25 (1.74 to 4.75) | 2.74 (1.87 to 3.61) |
|  | Pakistani | * | 2.6 (.298 to 4.91) |
|  | Bangladeshi | 0 (0 to 0) | 1.71 (-.397 to 3.82) |
|  | Other Asian | * | 1.95 (.68 to 3.22) |
|  | Caribbean | 2.84 (.922 to 4.75) | 2.32 (.903 to 3.74) |
|  | Black African | 3.74 (.253 to 7.23) | 2.58 (1 to 4.16) |
|  | Chinese | * | 3.04 (.527 to 5.56) |
|  | Other | 2.23 (.00355 to 4.45) | 2.65 (1.06 to 4.23) |
|  | **Age groups** |  |  |
|  | 25-49 | 1.86 (1.21 to 2.5) | 2 (1.74 to 2.26) |
|  | 50-59 | 1.44 (1.05 to 1.82) | 1.24 (1.08 to 1.4) |
|  | 60-69 | .867 (.579 to 1.16) | .86 (.722 to .997) |
|  | 70-79 | .458 (.164 to .752) | .635 (.473 to .798) |
|  | 80+ | * | .518 (.189 to .847) |
|  |  |  |  |
| R^2^ explained variation | **overall** | **58.1 (54.8 to 61.5)** | **54.6 (52.8 to 56.4)** |
|  | **Ethnic groups** |  |  |
|  | White | 56.1 (52.5 to 59.7) | 52 (50 to 53.9) |
|  | Indian | 71.5 (52.5 to 90.5) | 64.1 (49.4 to 78.8) |
|  | Pakistani | * | 60.1 (22.1 to 98.1) |
|  | Bangladeshi | * | * |
|  | Other Asian | 66.5 (10.1 to 123) | 47.1 (14.2 to 79.9) |
|  | Caribbean | 64.8 (33.8 to 95.7) | 56 (26 to 86) |
|  | Black African | 75.8 (35.1 to 117) | 60.6 (31.2 to 89.9) |
|  | Chinese | * | 68 (31.8 to 104) |
|  | Other | 53.8 (2.38 to 105) | 61.7 (31.6 to 91.8) |
|  | **Age groups** |  |  |
|  | 25-49 | 45.2 (27.9 to 62.4) | 48.8 (42.3 to 55.2) |
|  | 50-59 | 33 (21.1 to 44.8) | 26.7 (21.7 to 31.8) |
|  | 60-69 | 15.2 (6.65 to 23.8) | 15 (10.9 to 19.1) |
|  | 70-79 | * | 8.79 (4.68 to 12.9) |
|  | 80+ | * | * |

* Are cells with too few observations to be estimated reliably

**Supplementary table 7c Performance of the CanPredict risk algorithm in prediction of oesophageal squamous carcinoma 10-year risk in the QResearch validation cohort**

|  |  | **women** | **men** |
| --- | --- | --- | --- |
| *statistic* |  | *Mean (95CI)* | *Mean (95CI)* |
| Harrell's C | **overall** | **.868 (.856 to .881)** | **.854 (.84 to .868)** |
|  | **Ethnic groups** |  |  |
|  | White | .864 (.858 to .869) | .853 (.847 to .859) |
|  | Indian | .87 (.816 to .925) | .935 (.917 to .954) |
|  | Pakistani | .643 (.454 to .832) | .909 (.854 to .964) |
|  | Bangladeshi | .942 (.92 to .963) | .497 (.402 to .593) |
|  | Other Asian | .83 (.752 to .907) | .846 (.78 to .912) |
|  | Caribbean | .853 (.823 to .882) | .835 (.798 to .871) |
|  | Black African | .949 (.919 to .979) | .86 (.833 to .888) |
|  | Chinese | .984 (.968 to 1) | .882 (.779 to .985) |
|  | Other | .974 (.961 to .987) | .882 (.846 to .918) |
|  | **Age groups** |  |  |
|  | 25-49 | .819 (.728 to .909) | .805 (.722 to .889) |
|  | 50-59 | .708 (.65 to .766) | .679 (.625 to .733) |
|  | 60-69 | .669 (.622 to .717) | .7 (.649 to .75) |
|  | 70-79 | .588 (.537 to .64) | .585 (.521 to .648) |
|  | 80+ | .569 (.473 to .665) | .507 (.373 to .64) |
|  |  |  |  |
| Royston’s D statistic | **overall** | **2.4 (2.26 to 2.55)** | **2.2 (2.04 to 2.35)** |
|  | **Ethnic groups** |  |  |
|  | White | 2.33 (2.18 to 2.49) | 2.14 (1.97 to 2.3) |
|  | Indian | 2.8 (1.7 to 3.9) | 2.67 (1.27 to 4.07) |
|  | Pakistani | * | 2.61 (.0297 to 5.2) |
|  | Bangladeshi | 3.1 (1.21 to 4.99) | * |
|  | Other Asian | 2.75 (1.13 to 4.37) | 2.7 (1.03 to 4.37) |
|  | Caribbean | 2.13 (1.14 to 3.11) | 2.19 (1.3 to 3.09) |
|  | Black African | 3.12 (.957 to 5.27) | 2.31 (.603 to 4.02) |
|  | Chinese | * | * |
|  | Other | 3.4 (1.6 to 5.2) | 2.19 (.657 to 3.72) |
|  | **Age groups** |  |  |
|  | 25-49 | 3.45 (2.9 to 4.01) | 2.57 (2.08 to 3.06) |
|  | 50-59 | 1.54 (1.21 to 1.87) | 1.09 (.801 to 1.37) |
|  | 60-69 | 1.09 (.828 to 1.35) | 1.18 (.913 to 1.44) |
|  | 70-79 | .493 (.225 to .761) | .466 (.124 to .808) |
|  | 80+ | * | * |
|  |  |  |  |
| R^2^ explained variation | **overall** | **58 (55 to 61)** | **53.5 (50.1 to 57)** |
|  | **Ethnic groups** |  |  |
|  | White | 56.5 (53.3 to 59.8) | 52.1 (48.3 to 55.9) |
|  | Indian | 65.1 (47.3 to 83) | 62.9 (38.2 to 87.5) |
|  | Pakistani | * | 60.5 (12 to 109) |
|  | Bangladeshi | 69.6 (44.1 to 95.1) | * |
|  | Other Asian | 63.9 (35.8 to 91.9) | 63.1 (33.7 to 92.4) |
|  | Caribbean | 51.8 (28.7 to 74.9) | 53.4 (33 to 73.7) |
|  | Black African | 69.2 (38 to 100) | 55.9 (19 to 92.7) |
|  | Chinese | * | * |
|  | Other | 73.2 (52.1 to 94.3) | 52.7 (16.4 to 89) |
|  | **Age groups** |  |  |
|  | 25-49 | 74 (67.8 to 80.2) | 61.2 (52.2 to 70.2) |
|  | 50-59 | 36.1 (26.1 to 46.1) | 22 (13 to 31) |
|  | 60-69 | 22.1 (13.8 to 30.4) | 24.8 (16.5 to 33.2) |
|  | 70-79 | * | * |
|  | 80+ | * | * |

* Are cells with too few observations to be estimated reliably

**Supplementary Table 8 Calibration slope and intercept for 10-year risk of oesophageal cancer using CanPredict in the men and women aged 25-84 in the QResearch validation cohort overall, by ethnic group and age.**

|  | **QResearch** | **QResearch** | **QResearch** | **QResearch** |
| --- | --- | --- | --- | --- |
|  | **women** | **women** | **men** | **men** |
|  | **Calibration slope** | **Calibration intercept** | **Calibration slope** | **Calibration intercept** |
|  |  |  |  |  |
| overall | .992 (.944 to 1.04) | -.00816 (-.0556 to .0393) | .966 (.934 to .998) | -.034 (-.0658 to -.00218) |
|  |  |  |  |  |
| <70 years | 1.07 (.996 to 1.14) | .0661 (-.00367 to .136) | 1.01 (.97 to 1.05) | .0105 (-.0296 to .0506) |
| 70+ years | .651 (.513 to .789) | -.349 (-.487 to -.211) | .65 (.531 to .768) | -.35 (-.469 to -.232) |
|  |  |  |  |  |
| White | 1.01 (.961 to 1.06) | .0119 (-.039 to .0628) | 1 (.966 to 1.04) | .00097 (-.0337 to .0356) |
| Indian | 1.02 (.628 to 1.42) | .0228 (-.372 to .418) | 1.16 (.857 to 1.47) | .164 (-.143 to .472) |
| Pakistani | 1.07 (.255 to 1.89) | .0725 (-.745 to .89) | .91 (.475 to 1.35) | -.0901 (-.525 to .345) |
| Bangladeshi | 1.08 (.299 to 1.85) | .0767 (-.701 to .854) | .696 (.275 to 1.12) | -.304 (-.725 to .117) |
| Other Asian | 1.13 (.623 to 1.64) | .134 (-.377 to .644) | .915 (.438 to 1.39) | -.0854 (-.562 to .391) |
| Caribbean | 1.18 (.668 to 1.68) | .175 (-.332 to .682) | .836 (.543 to 1.13) | -.164 (-.457 to .13) |
| Black African | 1.04 (.347 to 1.74) | .0412 (-.653 to .736) | 1.05 (.241 to 1.85) | .0469 (-.759 to .853) |
| Chinese | 1.31 (-.128 to 2.75) | .312 (-1.13 to 1.75) | 1.32 (.314 to 2.32) | .315 (-.686 to 1.32) |
| Other | 1.22 (.627 to 1.8) | .216 (-.373 to .804) | .949 (.425 to 1.47) | -.0505 (-.575 to .474) |

1. Kunzmann AT, Thrift AP, Cardwell CR, et al. Model for Identifying Individuals at Risk for Esophageal Adenocarcinoma. *Clin Gastroenterol Hepatol* 2018;16(8):1229-36.e4. doi: 10.1016/j.cgh.2018.03.014 [published Online First: 20180317]

2. Wang QL, Ness-Jensen E, Santoni G, et al. Development and Validation of a Risk Prediction Model for Esophageal Squamous Cell Carcinoma Using Cohort Studies. *Am J Gastroenterol* 2021;116(4):683-91. doi: 10.14309/ajg.0000000000001094
