# Supplementary figures and images for: Development and validation of a novel risk prediction algorithm to estimate 10-year risk of oesophageal cancer in primary care: prospective cohort study and evaluation of performance against two other risk prediction models
Source: Lancet Reg Health Eur. 2023 Aug 14;32:100700. doi: 10.1016/j.lanepe.2023.100700 (PMC10450987; doi:10.1016/j.lanepe.2023.100700)

# QResearch women

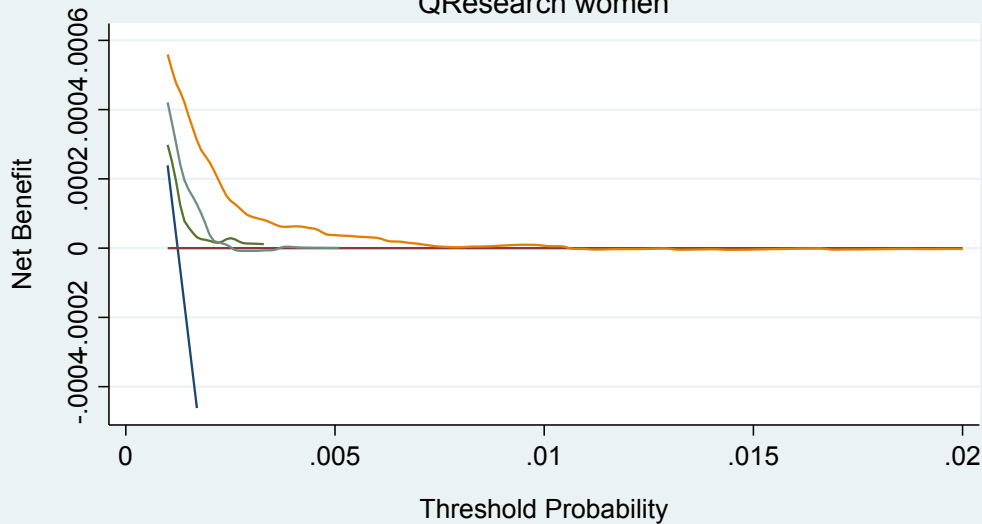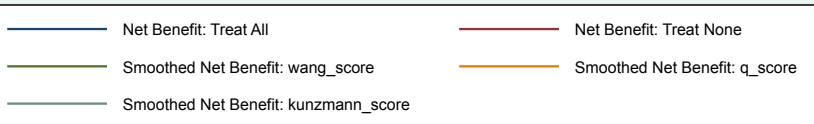

Supplement: Supplementary Figure S18 [file mmc3.pdf]
