# Supplementary material for: Development and validation of a novel risk prediction algorithm to estimate 10-year risk of oesophageal cancer in primary care: prospective cohort study and evaluation of performance against two other risk prediction models
Source: Lancet Reg Health Eur. 2023 Aug 14;32:100700. doi: 10.1016/j.lanepe.2023.100700 (PMC10450987; doi:10.1016/j.lanepe.2023.100700)
Supplement: Supplementary Figure S19 [file mmc4.pdf]

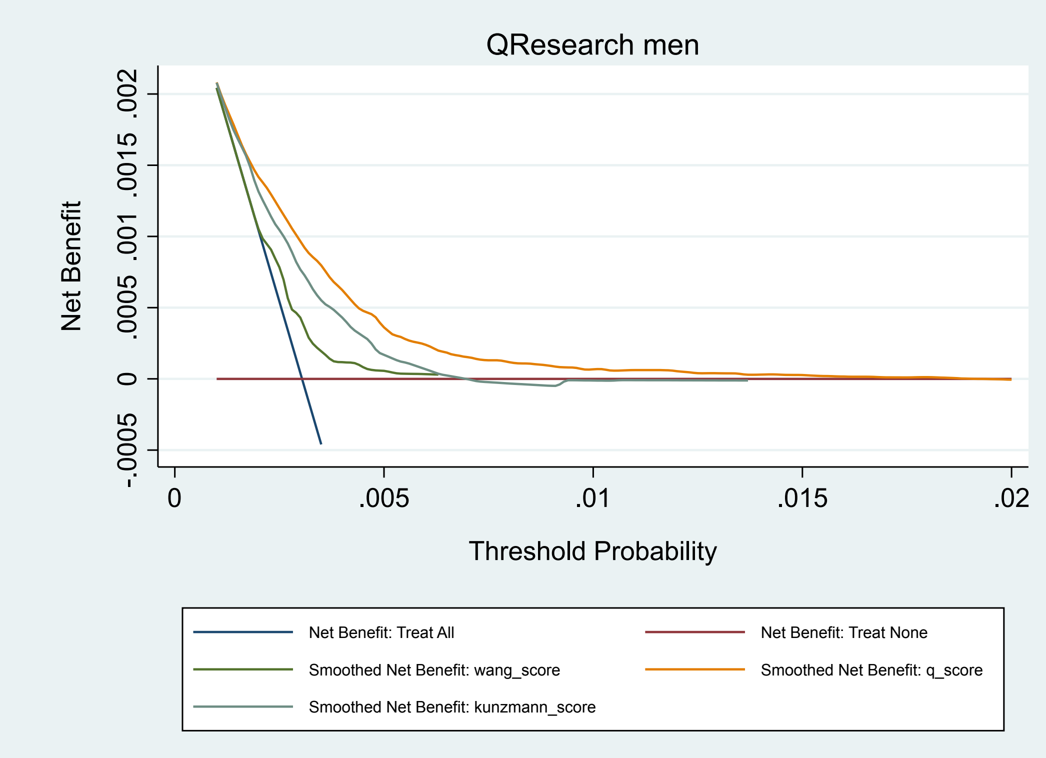
QResearch men
Net Benefit
-.0005
0
.0005
.001
.0015
.002
0
.005
.01
.015
.02
Threshold Probability
Net Benefit: Treat All
Net Benefit: Treat None
Smoothed Net Benefit: wang_score
Smoothed Net Benefit: q_score
Smoothed Net Benefit: kunzmann_score
